# Current Research on MoS_2_-Based Heterojunction Photocatalysts for Persistent Organic Pollutants Degradation

**DOI:** 10.3390/molecules30244727

**Published:** 2025-12-10

**Authors:** Luminita Isac, Cristina Cazan

**Affiliations:** 1 Product Design, Mechatronics and Environmental Department, Transilvania University of Brasov, 500036 Brasov, Romania; 2 Renewable Energy Systems and Recycling Research Center, Transilvania University of Brasov, 500036 Brasov, Romania

**Keywords:** MoS_2_-based photocatalysts, heterojunctions, photocatalysis, persistent organic pollutants, wastewater treatment

## Abstract

Currently, continuous population growth and unsustainable industrialization have caused ongoing water pollution, with harmful consequences for human health and the environment. Persistent organic pollutants (dyes, active pharmaceutical compounds, pesticides, etc.) are discharged into water from various industrial, agricultural, and domestic activities. Therefore, wastewater treatment through sustainable technologies is imperative, representing a great and real challenge for worldwide research. Photocatalysis, an innovative and green technology, uses advanced oxidation processes in the presence of a photocatalyst, usually a semiconductor with expanded light absorption ability and high conductivity for photogenerated charge carriers. Molybdenum disulfide (MoS_2_) is an n-type semiconductor with different morphologies, variable bandgap energies (Eg = 1.1–2.63 eV), and numerous applications. Although pristine MoS_2_ exhibits special structural and optoelectronic properties, its photocatalytic activity can be further improved through various strategies, and constructions with the heterojunctions construction with other semiconductors being frequently pursued. This review extensively studies the recent research (the last 4 years) on MoS_2_ and MoS_2_-based heterojunction (I-type, II-type, Z-scheme, S-scheme) photocatalysts for degrading organic contaminants under simulated and sunlight irradiation in wastewater treatment. Even if in a relatively short time (a few years) valuable studies have been reported on this topic, there are still numerous challenges facing future research.

## 1. Introduction

The improper disposal of wastewater in water bodies (rivers, lakes) represents a real and current problem, both for aquatic environments and for human life and development. Wastewater from various industrial, household, hospital, and agricultural activities is loaded with toxic, non-biodegradable, and recalcitrant organic pollutants (persistent organic pollutants, POPs), such as synthetic dyes [1,2,3,4,5,6], pharmaceutically active compounds (PhACs), personal care products, microplastics [6], pesticides [6,7], and nitroaromatic compounds [4].

Synthetic dyes are discharged in surface water from industrial sectors such as textiles, dyeing, paper, leather, rubber, cosmetics, plastics, food, etc. It is estimated that wastewater from the textile industry contributes approximately 20% to global industrial water pollution, due to manufacturing processes (washing, dyeing, and finishing) that consume large amounts of water, chemicals, and energy [6]. As an example, 10–15% of Methylene Blue (MB), the most widely used dye in the textile industry, is not adsorbed into the textile fibers but released with industrial effluents [8]. In the case of Indigo Carmine (IC) dye used in denim clothes manufacturing, approximately 10–25% is lost during the dyeing process via discharge into industrial water [9].

Similar to dyes, pharmaceutically active compounds (PhACs), including antibiotics (tetracyclines, fluoroquinolones), antiviral drugs, antidepressants, analgesics, hormones, etc., are released in water causing significant threats on public health and environment, especially to aquatic ecosystems [10]. Antibiotics in the environment can cause a multitude of problems by supporting the widespread development of resistance to antimicrobial products; thus, their removal is urgently needed.

Nitroaromatic compounds (NACs) are obtained in large quantities due to their widespread use as raw materials in the manufacture of dyes, pharmaceuticals, cosmetics, pesticides, fungicides, explosives, plastics, solvents, etc. Unfortunately, NACs are continuously discharged into the environment, contaminating water, soil, and air; the treatment processes used to their removal have proven to be less efficient and sustainable [4,11].

To maintain permissible concentration limits of pollutants discharged into surface and groundwater, wastewater treatment is required [12]. The available traditional wastewater treatment methods (flocculation, sedimentation, filtration, etc.) can achieve the removal of only small amounts of contaminants, while advanced technologies, e.g., adsorption, biodegradation, membrane separation, nanofiltration, electrocatalysis, ozonation, and photocatalysis, are most efficient for wastewater purification [12,13].

Most of these techniques do not successfully remove all pollutants from wastewater independently; therefore, innovative hybrid technologies have been developed, with improved process efficiency, stability, and sustainability. Examples of hybrid techniques reported in literature [14,15,16] are (1) combined ozonation with coagulation–flocculation and electrochemical oxidation (ECO), (2) adsorption on activated carbon and Fenton oxidation (FO), (3) photocatalysis combined with membrane processes, and (4) photocatalysis and biodegradation for the removal of bio-recalcitrant pharmaceuticals (antibiotics) from wastewater.

Photocatalysis is one of the simplest, economically feasible, and eco-friendly methods used for the complete mitigation of organic contaminants from wastewater effluents. This versatile technology harnesses solar energy to decompose the organic pollutants in wastewater, by advanced oxidation processes (AOPs), into harmless and controllable inorganic compounds such as CO_2_ and H_2_O. Recently, photocatalysis was recognized as a promising green technology with minimal risk of secondary environmental pollution [4,17,18]. However, photocatalysis is prone to some limitations, such as the rational development of new pathways to improve process yields and accelerate the transition from laboratory-scale to industrial applications [19].

In photocatalysis, a semiconductor material, the photocatalyst, absorbs light (natural, artificial) with an energy higher than its bandgap; as a result, its energy level increases, promoting the formation of energy-rich electron–hole pairs with active roles in photochemical reactions (reduction and oxidation). Therefore, in addition to a suitable bandgap energy for visible light absorption, chemical and physical stability, non-toxicity, availability, and low cost are important requirements to consider when selecting a semiconductor as the photocatalyst. Along with photocatalytic activity and stability, the selectivity is an important property required for a high-efficiency photocatalyst. The selectivity mainly depends on the photocatalyst’s design (band structure, surface active sites) and operating conditions (light absorption, pH, temperature, sacrificial agents). Strategies used to enhance selectivity for certain reaction pathways include photocatalyst synthesis and controlling the photocatalytic process, but also engineering the photocatalyst by doping, heterojunction construction, etc. [20]. Thus far, a variety of photocatalysts have been developed, such as the following:

- Metal oxides: TiO_2_ [17,21,22], ZnO [23,24], WO_3_ [25], SnO_2_ [26], etc.;
- Metal sulfides: ZnS [27], CuS [28], CdS [29,30], WS_2_ [31], In_2_S_3_ [32], MoS_2_ [33,34,35], etc;
- Carbon-based materials: carbon nanotubes (CNT) [36], graphene oxide (GO), reduced graphene oxide (rGO) [37,38], graphitic carbon nitride (g-C_3_N_4_) [39,40], carbon organic frame (COF) [41], etc.;
- Advanced materials: metal organic frame (MOF) [4], layered triple hydroxide (LTH) [9], etc.

As an important member of the class of transition metal dichalcogenides (TMDs), MoS_2_ is a noble metal-free, earth-abundant n-type semiconductor with variable bandgap (1.1–2.63 eV), high electronic mobility, good thermal stability, non-toxicity, water insolubility (as bulk), mechanical strength, remarkable flexibility, and quite low cost [18,41].

In addition, MoS_2_ can be synthesized by simple techniques, e.g., hydrothermal, solvothermal, ultrasonic, etc., and its structural and optoelectronic properties can be adjusted by controlling the synthesis conditions: precursors type, concentrations and molar ratios, nature and concentration of added solvents and/or surfactants, pH, reaction time, etc.

Currently considered a material with unique properties, MoS_2_ applications are various, including optoelectronic and energy storage devices, dry lubricants, sensors and biosensors, solar cells, biomedicine, etc. [42,43]. As photocatalysts, MoS_2_-based semiconductor materials are used in air [44,45] and wastewater [1,2,33,34,35] treatment, H_2_ production via water splitting [46,47,48,49], CO_2_ photoreduction to CH_4_, CO, CH_3_OH, and C_2_H_5_OH fuels [50,51,52], and organic synthesis [53].

However, the photooxidative capacity of pure MoS_2_ photocatalyst is somewhat limited due to its insufficient ability to generate highly reactive oxidative HO• radicals responsible for the degradation of organic pollutants [41]. To prevent this inconvenience, several strategies have been developed, such as crystal phase and edge engineering [10], metal/non-metal doping [10,49], noble metals (Au, Ag, Pt, Pd) deposition [54], sacrificial agents addition [50], and heterojunction construction [10,41,48,49,55]. MoS_2_-based heterojunctions prevent charge carrier recombination, optimizing light absorption over a wider spectral range. Thus, the development of new photocatalytically efficient, stable, and cost-effective heterostructure photocatalysts still remains a major challenge for researchers worldwide [56].

Thus far, numerous and varied MoS_2_-based photocatalysts with environmental remediation applications, especially in wastewater treatment, have been reported. This review intends to be a broad overview based on up-graded literature from the last 3–4 years related to the development of MoS_2_-based photocatalysts used for the degradation of different organic pollutants (e.g., organic dyes, pharmaceutical active compounds, phenol and phenolic compounds, pesticides) under simulated and natural sunlight irradiation.

This study highlights the photocatalytic performances of pristine MoS_2_ and MoS_2_ heterojunction photocatalysts in the degradation of persistent organic pollutants, for possible future improvements in their efficiency in wastewater treatment. Moreover, the construction of MoS_2_ heterojunctions with suitable semiconductors (metal oxides, metal sulfides, carbon-based material, MOF, LTH) has been comprehensively presented as an efficient strategy for enhancing MoS_2_ photocatalytic activity. We also point out current challenges and perspectives in developing MoS_2_-based heterostructure photocatalysts for large-scale wastewater treatment.

## 2. MoS_2_ as Photocatalyst

Considering its unique structure (crystalline phase and morphology versatility), special electronic, optic, magnetic, and mechanical properties, MoS_2_ is recognized as a promising material with various applications such as environmental remediation (photodegradation of organic and inorganic pollutants from air and wastewater) [1,2,33,34,35,44,45,46], electrochemical capacitors, ultra-low-leakage dynamic memory devices, optoelectronic devices (phototransistors, biosensors), solar cells, energy storage devices (Li-ion and Na-ion batteries, supercapacitors) [42,43], H_2_ production via low-cost photocatalytic water splitting [46,47,48], CO_2_ photoreduction to C1 (CO, HCOOH, HCHO, CH_3_OH, CH_4_), and C2 (C_2_H_4_, C_2_H_5_OH) fuels [50,51,52]. In addition to air and wastewater treatment, MoS_2_-based semiconductor materials are used in organic syntheses, such as nitroarene reduction, hydrodesulfurization, miscellaneous reactions, and the conversion of biomass into commercially valuable products: biofuels, phenolic compounds, and tar [53]. In terms of biomedical applications, a comprehensive review [42] reported that MoS_2_-based nanomaterials have key roles in drug-resistant bacteria destruction, photothermal therapy, and drug delivery.

Over time, various methods were used for MoS_2_ preparation, depending on the properties (crystal structure, morphological, optoelectronic, etc.) required for different applications. These properties can be controlled by selecting a simple, rapid, environmentally friendly, and low-cost synthesis method that can be easily adjusted according to the working conditions. However, every method has its own advantages and limitations.

In the most recent reports [10,43,57], two main approaches to MoS_2_ synthesis methods are related: (a) top-down, involving the reduction of bulk/multiple layered MoS_2_ to single/monolayer MoS_2_ by exfoliation (e.g., mechanical, chemical, liquid), and (b) bottom-up, consisting of aggregation of different type of MoS_2_ crystals to nanostructured layered structures (e.g., hydrothermal, solvothermal, chemical vapor deposition, photodeposition). Among these, the hydrothermal method is widely used for obtaining MoS_2_, due to its simple experimental set-up, cost-effectiveness, and ease of control through modifying the working parameters [58]. Thus, the convenient selection of the precursors type and concentration, solvent(s), stabilizing agent(s), pH, reaction temperature, and time have significant influence on the properties of prepared MoS_2_ [10]. Moreover, hydrothermal methods are frequently used to obtain hybrid materials or composites. In addition to the many advantages, some already mentioned, hydrothermal methods still present limitations, such as low yields and long reaction times, thus reducing their application on an industrial scale [59].

### 2.1. Structure

**Figure 1:**
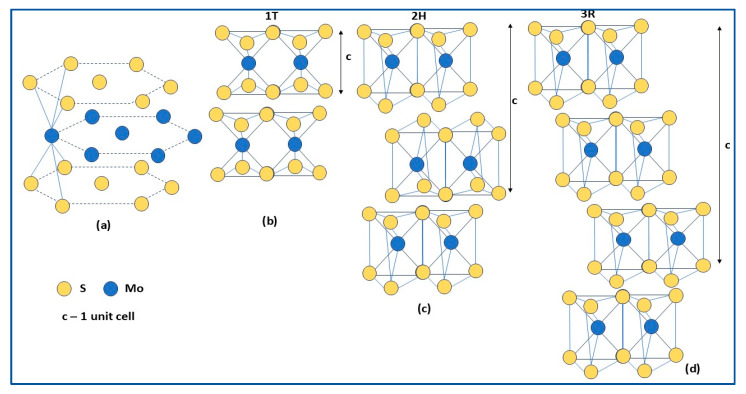
Crystal structure of (**a**) layered MoS_2_, (**b**) 1T-MoS_2_, (**c**) 2H-MoS_2_, and (**d**) 3R-MoS_2_ polymorphs (reproduced from Ref. [48]).

As a representative transition metal dichalcogenide (TMDC), MoS_2_ has a layered structure (Figure 1a), with each MoS_2_ layer consisting of stable S–Mo–S units separated by nanometric distances (~0.65 nm) and held together by weak Van der Waals forces. Each S–Mo–S layer has a sandwich structure formed by a hexagonal plane of Mo atoms (in the middle) and two hexagonal planes of S atoms (above and below) [41,48,58]. The crystalline MoS_2_ shows four polymorphs, namely 1H (1—one layer/unit cell, H—Hexagonal), 1T (1—one layer/unit cell, T—Tetragonal), 2H (2—two layers/unit cell, H—Hexagonal), and 3R (3—three layers/unit cell, R—Rhombohedral), differentiated by the stacking arrangement and coordination between the central Mo atom and surrounding S atoms. Even if 1H-MoS_2_ is the most stable polymorph, the three commonly structures are the following:

- 1T-MoS_2_, with a metastable octahedral structure composed of one S–Mo–S layer per unit cell, where Mo is exposed on the surface (Figure 1b); it could be stabilized by doping or by hybrid structures formation; it shows electrical behavior and relative hydrophilicity, therefore, it is more suitable for hydrogen production [48];
- 2H-MoS_2_, the most commonly used, has a thermodynamically stable trigonal prismatic structure (Figure 1c); it has semiconducting, hydrophobic, and photoluminescent properties, a narrow bandgap, and a larger specific surface area; hence, many active sites characterize this structure [42,53,58];
- 3R-MoS_2_ has a metastable structure with trigonal prismatic geometry (Figure 1d); it exhibits metallic behavior and, like the 1T polymorph, can easily transform into the 2H phase [53].

Although less studied, amorphous MoS_2_ has recently received considerable attention due to its various catalytic applications. Amorphous MoS_2_ nanoparticles (NPs) exhibit an extreme variety of arrangements of their structural units, with a higher number of unsaturated and deficient atoms concentrated on the MoS_2_ surface compared to crystalline MoS_2_ and, hence, greater (photo)catalytic performance [53].

### 2.2. Morphology

The MoS_2_ morphology, influencing the size, surface area, and surface energy of particles, significantly contributes to its performance in numerous applications, such as photocatalysis. Different morphologies of MoS_2_, i.e., nanoflowers, nanosheets, nanorods, nanoflakes, nano- and irregular microspheres, quantum dots (QDs), were reported so far, as shown in Table 1. The most common one is a nanoflower-like morphology with variable average size and petal thickness. Usually, MoS_2_ nanoflower structures display narrow bandgaps (1.31–2.4 eV) [2,3] and large surface areas, favoring the efficient absorption of visible light, thus enhancing their photocatalytic performance [10]. In this context, the photocatalytic activity of MoS_2_ with nanoflowers morphology, prepared by a simple hydrothermal method, was studied in the degradation of Rhodamine B (RhB) dye in concentrated sunlight irradiation (solar concentrator coupled optical fiber bundle) [33]. The prepared MoS_2_, consisting of flowers with 100 nm average size and petals several nanometers thick, with a calculated bandgap of 2.2 eV, showed good photocatalytic activity, degrading 67.4% of RhB dye after 120 min in concentrated sunlight, compared with only 39.9% degradation occurring under ordinary sunlight exposure. That means a 1.7 times faster degradation of RhB dye in concentrated sunlight, when the excess of photons generates more electron–hole pairs; hence, more hydroxyl (HO•) and superoxide (•O_2_^−^) radicals are formed, accelerating the dye degradation.

Layered MoS_2_ nanostructures were prepared via a hydrothermal method using Lawesson’s reagent (LR, C_14_H_14_O_2_P_2_S_4_) as a sulfur source and ammonium molybdate as a molybdenum source [2]. The morphology of MoS_2_ nanostructures, as a complex network formed by MoS_2_ layers trapped with other layers, shows similarities to graphene-based materials. The photocatalytic experiments (Table 1) on MB and Crystal Violet (CV) dyes, carried out under natural sunlight and UV lamp, revealed that layered MoS_2_ degraded 71% of MB and 57% of CV, respectively, and almost 82% of MB and 73% of CV, respectively, within 90 min. The photocatalytic activity of the MoS_2_-layered nanostructure was more enhanced (a) for MB degradation than CV, possibly due to the better electron–hole separation and the less complex structure of MB compared to that of CV, and (b) in UV light illumination compared with natural sunlight, limiting its use in natural environments [2].

### 2.3. Electronic Properties

The electronic properties of MoS_2_ were investigated using different Density Functional Theory (DFT) aspects and based on density functional theory and time-dependent density functional perturbation theory (TDDFPT) [60]. Based on Density Functional Theory (DFT) calculations in bulk MoS_2_, the d orbitals on molybdenum atoms, positioned in the center of the S–Mo–S structure, are assigned to the K-point in the minimum conduction band (CBM), and the antibonding p_z_ orbitals of sulfur occupied point Γ in the maximum valence band (VBM). Accordingly, bulk MoS_2_ (containing at least 10 layers) showed a small indirect bandgap (1.3 eV), insufficient to induce photocatalytic reactions and the separation of charge carriers. Decreasing the layers number, the interaction between Mo(4d)-S(3p) orbitals conducted to a new Γ position in the valence band maximum (VBM), with higher interlayer coupling capacity. As a result, the bandgap formed between the stable K-point in the CBM and new Γ position in the VBM increased to about 1.8–1.9 eV (monolayer MoS_2_). Thus, with decreasing the number of layers, the bandgap of MoS_2_ increases, allowing a broader absorption of light in the visible range. The bandgap variation with the number of layers is a unique property of MoS_2_, called the tunable bandgap, making it a promising semiconductor in optoelectronics and electronics applications [42,48,61,62,63]. According to literature (Table 1), the bandgap energies for pristine MoS_2_ with different morphologies vary from 1.1 eV for 2D MoS_2_ sheets [24] to 2.63 eV for MoS_2_ microstructures with an average diameter of ~50 μm [36].

In addition to variations in the number of layers, MoS_2_ semiconductor electronic properties could be tailored by quantum confinement effects, mechanical strain application, and doping. Quantum confinement refers to significantly changing in electronic properties of a semiconductor due to size reduction to ultra-small dimensions (quantum dots, QDs), resulting in a discrete density of states and a bandgap that varies inversely with the size of the QDs. The emerging effects of quantum size, such as increased active surface area and strong electronic interactions, contribute to the rapid transfer of charge carriers and improved electron–hole separation, thus reducing the charge recombination rate, which results in improved photocatalytic activity of the photocatalyst [64]. Mechanical deformation has the effect of changing the bandgap from direct to indirect, making the semiconductor behavior of MoS_2_ become metallic. MoS_2_ exhibits n-type (doped with Cu, Cr, Sc), p-type (doped with Zn, Ni), or even both semiconducting properties by doping with Ti, depending on Ti concentrations and doping sites [65].

### 2.4. Optical Properties

One of the most important properties of a photocatalyst used in pollutant degradation in wastewater is the ability to absorb solar energy. Since the wavelength of absorbed light and the bandgap energy are inversely proportional, photons with a higher wavelength and lower energy than the semiconductor material bandgap energy are not absorbed by it. The absorption coefficient quantifies the ability of a material to absorb energy, with higher absorption coefficients characterizing an absorbent material, while lower coefficients correspond to transparent or reflective materials. Using the light attenuation model from the Raman and AFM measurements, Kwak [66] reported an absorption coefficient of 2.8 × 10^6^ cm^−1^ for the thin MoS_2_ flakes, which is slightly higher than those of MoS_2_ monolayer, 1.5 × 10^6^ cm^−1^. The optical properties, analyzed via photoluminescence (PL) measurements, UV–VIS, and Raman spectroscopy, showed that the absorption coefficient of MoS_2_ is higher in the range of 400–700 nm, demonstrating the absorption of light by pure MoS_2_ in both UV and VIS ranges [48,62]. For example, MoS_2_ monolayer was reported [67] to absorb 23%, 6%, and 7% of the incident light at 432 nm, 617 nm, and 664 nm wavelengths, respectively. These absorption amounts seem to be promising compared to the MoS_2_ thickness; however, to design an efficient MoS_2_-based absorber, these values need to be increased. A strategy in this way could involve using stacks of layers in the form of photonic crystals or quasi-photonic crystals.

The optoelectronic properties of MoS_2_ can be tailored by (a) varying the bandgap and transitions induction between direct and indirect bandgaps, causing strains; (b) doping, thus modifying carrier concentration and band alignment; and (c) introducing defects (midgap states) such as sulfur vacancies. Fine-tuning of structural, electronic, and optical properties allows the use of MoS_2_ in specific applications [62].

### 2.5. MoS_2_ and Metal-Doped MoS_2_ Photocatalysts

**Figure 2:**
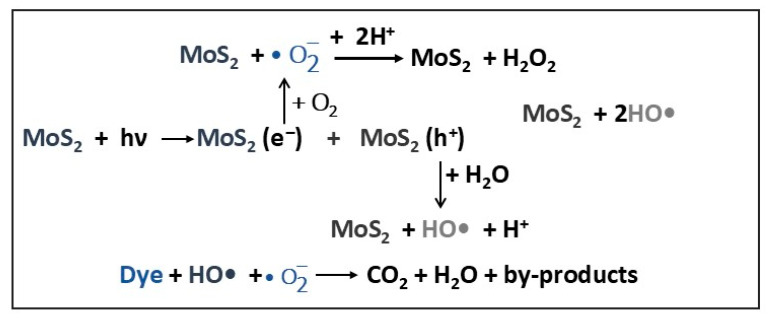
The degradation mechanism of dyes from industrial wastewater by assisted surfactant-MoS_2_ photocatalyst.

Farooq et al. [5] synthesized MoS_2_ nanostructures via a hydrothermal method using four different surfactants: urea (UREA), polyvinylpyrrolidone (PVP), cetyltrimethylammonium bromide (CTAB), and oxalic acid (OA). The prepared MoS_2_ samples were noted as M-UREA, M-PVP, M-CTAB, and M-OA, respectively. The differences between the morphologies of pure MoS_2_ and those of surfactant-assisted MoS_2_ are significant. Thus, if the pure MoS_2_ structure consists of rods, sheets, and other irregular shapes (average sizes of 70 nm) agglomerated, the surfactant-assisted MoS_2_ structures have uniformly distributed nanorods, with a smaller diameter (25 nm average size in M-UREA) and length. This size decrease, of almost three times, was explained by the formation of UREA (non-ionic) surfactant macromolecular hydrophilic film on the surface of MoS_2_ nanoparticles, which amplified repulsions, slowing down the nucleation process and restraining the agglomeration of prepared MoS_2_ particles. As a result, M-UREA has a higher specific surface area (45 m^2^/g) compared to pure MoS_2_ (29 m^2^/g), indicating the presence of mesoporous structures. The bandgap energies are quite close, in the range of 1.96 (M-UREA)–2 eV (MoS_2_), the slight reduction in the bandgap values being attributed to surface defects induced by surfactants during the photocatalyst synthesis process. The PL measurements showed that M-UREA had the lowest intensity emission spectrum among all other samples, while pure MoS_2_ exhibited the highest photoluminescence intensity, indicating low transfer and high recombination rate of charge carriers. The photocatalytic activity of MoS_2_ and surfactant-based MoS_2_ was tested in degrading pollutants from industrial wastewater obtained from the leather field industry, under visible light irradiation for 180 min (Table 1). The results confirmed that the highest efficiency in dyes photodegradation was demonstrated by M-UREA (59%), compared to the other samples (e.g., 45% for pure MoS_2_), due to its properties: lowest bandgap energy (1.96 eV), optimal nanorods morphology with high BET (Brunauer–Emmett–Teller) surface area (45 m^2^/g), higher transfer, and reduced recombination rate of photogenerated charge carriers [5]. The mechanism proposed by the authors for degradation of dyes in leather industry wastewater using surfactant-based MoS_2_ is schematically presented in Figure 2.

VIS light radiation on the VB and CB of MoS_2_ catalyst photoinduced generated electron–hole pairs that transferred to the layered MoS_2_ surface. The photogenerated electrons (e^−^) reduce dissolved oxygen from water to superoxide anion radicals (•O_2_^−^), which further react with H^+^ ions and form H_2_O_2_. Simultaneously, the photogenerated holes (h^+^) react with H_2_O molecules adsorbed on the surface of layered MoS_2_, resulting in hydroxyl radicals (HO•). The resulting radicals are highly reactive species that oxidize and decompose dye molecules from industrial wastewater into CO_2_, H_2_O, and small amounts of other products (byproducts).

Currently, obtaining nanomaterials via green synthesis is of particular interest for supporting environmental sustainability, including their uses in various industrial and medical fields. By combining MoS_2_ with botanical extracts, hybrid heterostructures were obtained as result of the arrangement of MoS_2_ nanosheets and the distribution of plant extracts. These heterostructures can significantly improve light absorption and charge carrier separation, crucial requirements in photocatalysis applications. Sathishkumar et al. [35] reported the preparation of pure and biosynthesized MoS_2_ nanoparticles using extracts from the bark of Ficusreligiosa L (FR) and leaves of ZiziphusjujubeL (ZJ). The prepared materials displayed a hexagonal phase structure (2H-MoS_2_) with the crystalline size decreasing from approximately 13 nm (pure MoS_2_) to 6 nm (MoS_2_-ZJ) and 4 nm (MoS_2_-FR), respectively, indicating the influence of plant extract on MoS_2_ crystals formation. For both pure and biosynthesized MoS_2_ NPs, the surface morphology consisted of nanoflakes with a spherical size distribution and a wide variety of sizes, varying from nanometers to micrometers. As is shown in Table 1, the FR and ZJ plant extracts caused changes in surface area; as a result, BET surface area increased from 79 m^2^/g (MoS_2_ NPs) to 134 m^2^/g (biosynthesized MoS_2_ NPs, MoS_2_-ZJ). The bandgap energy (Eg) decreased in the same way, from 2.37 eV (MoS_2_) to 2.03 eV (MoS_2_-ZJ), due to the encapsulation of oxygen and carbon molecules from the plant extract into the MoS_2_ crystal structure. Although all the samples showed remarkable oxytetracycline (OTC) antibiotic photodegradation (over 95%) after 120 min in VIS-light irradiation, the highest degradation efficiency (99%) of MoS_2_:FR catalyst, within 100 min, was attributed to the significant influence of FR plant extract on the average crystalline size (4 nm), suitable bandgap energy (2.21 eV), and specific surface area (121 m^2^/g) [35].

One of the strategies to improve the photocatalytic activity of MoS_2_ is doping with transition metals or non-metals, when the number of active sites increases. This can be easily achieved by tailoring the MoS_2_ morphology, which, in turn, can be controlled by the synthesis conditions. Among transitional metal dopants, Ag NPs are considered appropriate for enhancing the MoS_2_ photocatalytic efficiency due to their low cost, non-toxicity, good chemical stability, and excellent optical properties. By doping MoS_2_ with Ag NPs, the sulfur atoms are slightly displaced by Ag atoms and the new active sites formed increase the electrical conductivity of MoS_2_, hence the photocatalytic properties [8,32]. Nanoparticles of Ag-doped MoS_2_ (Ag-MoS_2_) were synthesized by a hydrothermal method, varying the dopant (Ag) concentration, to evaluate the photocatalytic activity in degrading MB under UV light [32] or in presence of a reducing agent (NaBH_4_) [8].

Pure MoS_2_ exhibited a sheet-like structure, containing individual slightly curved or twisted monodisperse sheets, a common morphology in MoS_2_ synthesized by hydrothermal reactions. After doping with Ag NPs, a change in MoS_2_ morphology was observed: the size and thickness of sheets decreased and distinct particles of Ag were randomly distributed to the surface of the MoS_2_ sheets. Even if it is expected that this morphology, displaying large specific areas and high numbers of active sites, significantly improves the photocatalytic process, the photodegradation of MB under UV light exposure increase was only 15% when 3%Ag-MoS_2_ is used as catalyst compared with MoS_2_ [33].

However, doping MoS_2_ with Ag NPs increased the crystallite size from approximately 7 nm (MoS_2_) to 9.8 nm (Ag-MoS_2_ with higher Ag concentration), while bandgap energy decreased from 2.35 eV (MoS_2_) to 1.55 eV (Ag-MoS_2_). Photocatalytic experiments performed using a reducing agent (NaBH_4_) showed (Table 1) that bare MoS_2_ displayed limited removal (40%) of MB dye within 20 min, while the Ag-doped sample (5%Ag-MoS_2_) degraded 74% of MB in 4 min. The differences in photocatalytic activity of bare and Ag-doped MoS_2_ were attributed to the added dopant concentration, the higher surface area and crystallinity, but also to the presence of an additional reducing agent [8].

Metallic Au NPs, acting as a dopant in MoS_2_ photocatalyst, contribute to improving its performance by diminished photogenerated electron/hole pair recombination through Schottky junctions formation and/or additional charge carriers generation [67]. It has been reported [68] that the integrated Au-MoS_2_ nanoflowers structure provided superior photocatalytic activity compared with bare MoS_2_ nanosheets, due to the sensitization of MoS_2_ nanoflowers with optical stimulation of plasmonic resonant Au NPs. In a typical photocatalytic experiment, 5 mg of photocatalyst prepared by a photochemical method was dispersed in 20 mL of MB dye aqueous solution (6 × 10^−6^ M) and exposed to direct light irradiation (photoreactor PR-2000) for 60 min. The MB dye photodegradation efficiency was almost double after doping MoS_2_ with Au NPs, i.e., an increase from 35% to 63%. The limited improvement in Au-MoS_2_ photocatalytic activity is due to the formation of a Schottky junction that favors the movement of photoexcited holes of MoS_2_ into electron-occupied Au states, causing charge recombination. To overcome this disadvantage, the Au-MoS_2_ structure was engineered by introducing a CuS capping layer. This strategy proved to be beneficial, as the MB photodegradation efficiency increased to 90.5% in the presence of MoS_2_-Au/CuS photocatalyst. The introduction of the p-type semiconductor CuS, besides adjusting the interfacial electrical barrier in the junction to prevent charge recombination, can act as a protective layer for Au NPs in direct contact with dye solutions, providing long-term sustainability of the photocatalyst [69].

**Figure 3:**
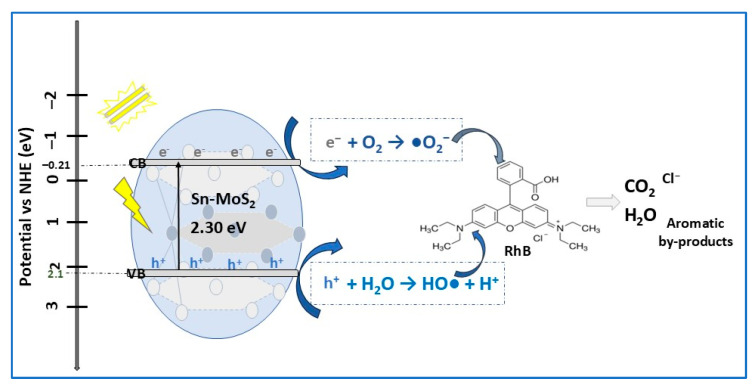
The photocatalytic degradation mechanism of RhB dye using Sn-doped MoS_2_ photocatalyst under VIS light irradiation.

Pristine MoS_2_ (P-MoS_2_) and Sn-doped MoS_2_ (D-MoS_2_) were prepared by the hydrothermal method. To improve photocatalytic properties, they were dried either directly in an oven (8 h, 75 °C) or through a freeze-drying process (lyophilization, 30 h, −50 °C, DL-MoS_2_) [3]. According to XRD analysis, the predominant crystalline phase in P-MoS_2_ was 2H-MoS_2_, along with some amounts of the 3R-MoS_2_ phase. By adding 2.5% of Sn, a shift in the main peak (100) towards a higher 2θ value was observed, thus confirming the successful infiltration of Sn into the MoS_2_ matrix (Sn doping). Both P-MoS_2_ and D-MoS_2_ samples showed a spherical flower-like morphology with differences in spherical flower diameter and interconnected nanosheet thickness. In contrast, lyophilized Sn-doped MoS_2_ (DL-MoS_2_) showed a nanoflake-like structure formed by very thin nanoflakes with a thickness of about 15–20 nm. Based on the N_2_ adsorption/desorption of P-MoS_2_, D-MoS_2_, and DL-MoS_2_ results, all the samples showed porous structures with a specific surface area of DL- MoS_2_ (127 m^2^/g) about 2 times that of D-MoS_2_ (65 m^2^/g) and 2.4 times that of P-MoS_2_ (53 m_2_/g). The bandgap energies of P-MoS_2_, D-MoS_2_, and DL-MoS_2_ decreased from 2.40 eV to 2.30 eV, indicating that the doping of MoS_2_ with Sn favored absorption in the visible light range. The photocatalytic experimental results (Table 1) show that the RhB dye was completely degraded in 40 min and in 30 min by P-MoS_2_ and D-MoS_2_, respectively. Using the DL-MoS_2_ catalyst, the RhB photodegradation process was faster, with total degradation achieved in 20 min, with 50% of RhB being degraded in only 5 min. In addition, DL-MoS_2_ demonstrated good photostability and reusability after four repeated cycles, proving to be a promising photocatalyst for dye degradation in industrial applications. The results of radical trapping experiments using 1, 4-benzoquinone (BQ), ammonium oxalate (AO) and tertiary butyl alcohol (TBA) as scavengers of the superoxide radicals (•O_2_^−^), and photo-induced holes (h^+^) and hydroxyl radicals (•OH), elucidated the RhB photodegradation mechanism by lyophilized Sn-doped MoS_2_ photocatalyst, as shown in Figure 3.

**Figure 4:**
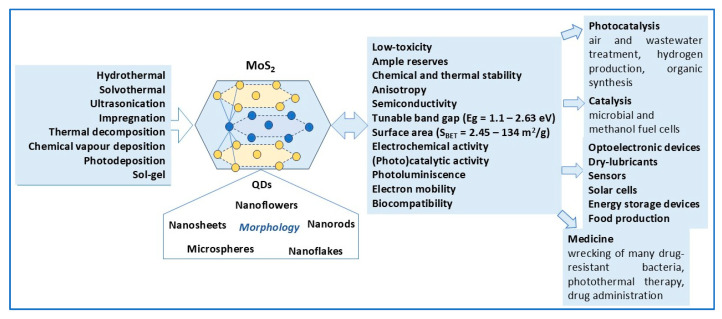
Summary of synthesis methods, specific properties, and applications of MoS_2_ nanostructure photocatalysts.

As a summary, Figure 4 shows methods for obtaining MoS_2_ photocatalysts, their specific properties (crystalline structure, morphology, bandgap energy, specific surface area), and applications.

## 3. MoS_2_-Based Heterojunction Photocatalysts

In contrast to the numerous advantages already mentioned, MoS_2_ may also present limitations, especially related to its (photo)catalytic activity, which can be considerably reduced due to the rapid recombination of photogenerated charge carriers. This is attributed to its low dispersive capacity as consequence of its hydrophobicity and low electrical conductivity. In addition, the lower number of active sites caused by the agglomeration of MoS_2_ layers, as result of Van der Waals interactions, negatively contributes to the photocatalytic activity of MoS_2_ [58]. To overcome this drawback, the following strategies have been proposed as effective solutions: (a) doping with heteroatoms (metals: Cu, Co, Fe, Ag, Mn; non-metals: P, N), activating MoS_2_ surface sites and narrowing the bandgap to enhance visible light wide adsorption [10,33,49]; (b) noble metals (Au, Ag, Pt, Pd) deposition and chemical adsorption on the MoS_2_ monolayer surface result in the MoS_2_ bandgap reduction due to impurity states forming in its bandgap [54]; (c) designing special architectures (2D, 3D) by crystal phase and edge engineering to increase the specific surface area [10]; (d) adding sacrificial agents with the role of either scavenging photogenerated holes or donating electrons to MoS_2_ in order to improve its photocatalytic activity [49,70]; (e) developing heterojunctions by coupling with one or more semiconductor(s), improving solar energy absorption, and activating surface redox reactions; in addition, the heterojunction interface can be tailored by selecting the appropriate method and synthesis conditions [10,48,62].

**Figure 5:**
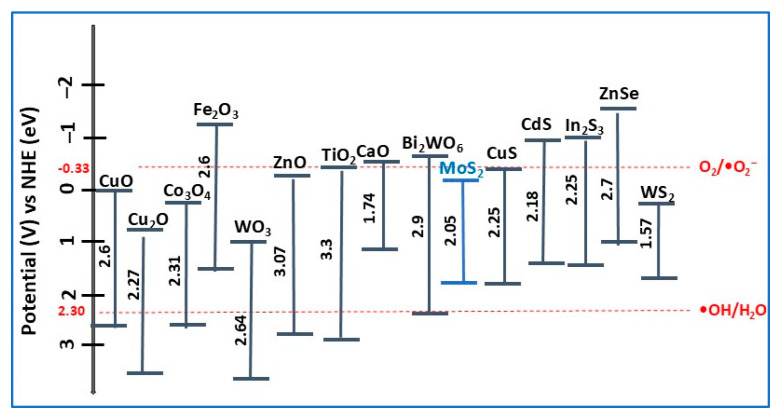
Band structures for photocatalysts mentioned in this review and their band edge potential [6] as a function of an NHE at pH = 7.

In heterostructure formation, MoS_2_ not only contributes to the increase in active sites for photocatalytic reactions but also accelerates charge carrier separation and transfer, preventing any recombination at the surface, which favors the photocatalytic degradation of pollutants [40,55]. Hence, the charge transfer mechanism can be modified by constructing different types of band alignments between the heterojunction components, improving the photocatalyst’s performance [48]. In MoS_2_-based heterojunctions, the bandgap is not a fixed value, depending on the heterojunction type, the other semiconductor bandgap, and the resulting band alignment. In Figure 5 are presented the bandgap values of MoS_2_ together with some other common photocatalysts and their corresponding redox potentials at pH = 7.

**Figure 6:**
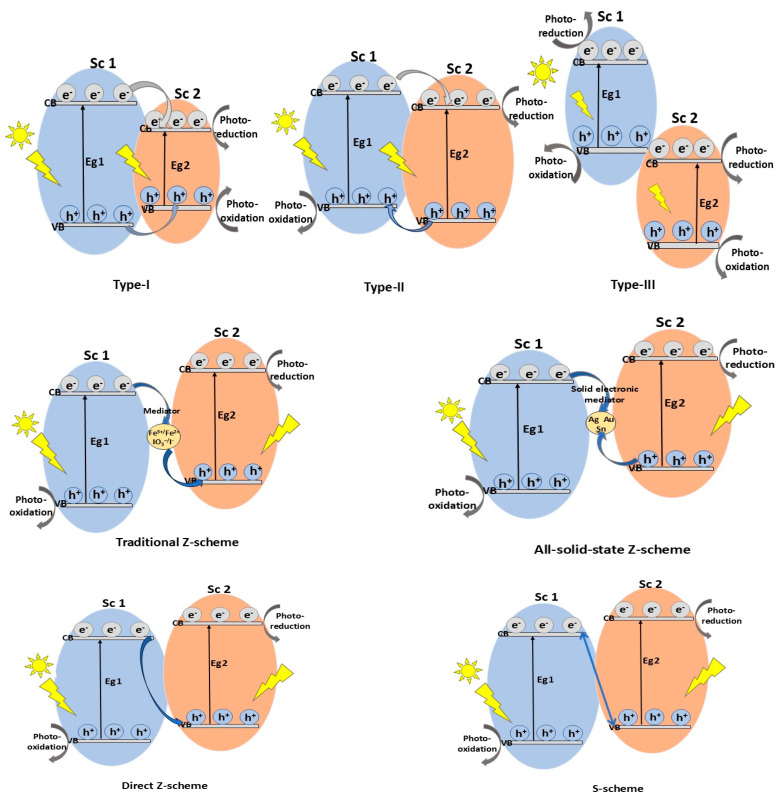
Heterojunctions: type-I, type-II, type-III, traditional Z-scheme, all-solid-state Z-scheme, direct Z-scheme, and S-scheme.

Depending on the conduction band (CB) and valence band (VB) alignment of the component semiconductors, the conventional heterojunctions formed in photocatalysts can be classified [10], according to Figure 6, as follows:

- **Type-I heterojunction**, with straddling bandgaps in which charge carriers transfer results in redox reactions occurring at the same semiconductor (Sc 2);
- **Type-II heterojunction**, with staggered bandgaps in which the CB and VB positions are at optimal levels, thus ensuring spatial charge carrier separation, enhancing photocatalytic performance compared to type-I; the oxidation and reduction reactions take place on Sc 1, with lower oxidation potential;
- **Type-III heterojunction**, with broken bandgaps in which there are no synergistic interactions between electrons and holes that would cause the separation of lower charge carriers, resulting in not thermodynamically favorable and stable photocatalytic reactions occurring compared with the type-II heterojunction.

Although these heterojunctions, especially type-II, have proven to be suitable for improving the charge carrier separation mechanism, there are still some shortcomings related to the charge carrier transfer between the two semiconductors, affecting their reduction (CB potential for Sc 1) and oxidation (VB potential for Sc 2) abilities [48]. To achieve good charge separation efficiency without compromising the redox capacity of the semiconductors, **Z-scheme heterojunction** photocatalysts have been developed with a charge mediator between the two semiconductors [62]. Under VIS light irradiation, the photogenerated carriers, electrons in the CB (Sc 2), and holes in the VB (Sc 1) combine forming strongly oxidative holes in the VB (Sc 2) and reductive electrons in the CB (Sc 1), inducing an electric field at the interface that accelerates electron–hole pair separation. In this heterojunction, the charge carrier transfer pathway resembles the letter Z and may involve a mediator to enhance transfer efficiency. Z-scheme photocatalysis can be described by three main mechanisms, shown in Figure 6.

Similar to the Z-scheme heterojunction, the **S-scheme heterojunction** has been proposed as a new, improved alternative to the type-II heterojunction, describing more clearly the photocatalytic mechanism. In a typical type-II heterojunction, photogenerated electrons and holes accumulate on the CB of Sc 2 (oxidation semiconductor) and the VB of Sc 1 (reduction semiconductor), resulting in weak redox ability. In contrast, in the S-scheme heterojunction, the CB of Sc 1 is occupied with the photogenerated electrons, while the VB of Sc 2 contains photogenerated holes, and useless photogenerated charge carriers are recombined, introducing a strong redox potential. Therefore, the path followed by charge transfer in the S-scheme mechanism is different and resembles a “Step” (from low CB to high CB), hence the name. In addition, the semiconductors of the S-scheme heterojunction can also be n-type or p-type, provided that both the CB and Fermi levels of the reduction semiconductor (Sc 2) are higher than those of the oxidation semiconductor (Sc 1) [71].

A summary of recent studies on MoS_2_-based heterojunction photocatalysts, prepared by various methods, and their photocatalytic performances in degrading persistent organic pollutants using different light sources is presented in Table 2.

Based on literature (Table 2), depending on several parameters related to experimental conditions, MoS_2_-based photocatalysts have been used in the degradation of different types of organic pollutants, particularly dyes, pharmaceuticals, and phenols. Due to their ability to absorb a broader spectrum of light, resulting in charge carrier separation, MoS_2_-based photocatalysts readily degrade a wide range of dyes, mainly MB, RhB, and MO. For the same dyes (RhB and MB), MoS_2_ ternary photocatalysts (α-Fe_2_O_3_/MoS_2_/g-C_3_N_4_, MoS_2_/Co_3_O_4_/Cu_2_O) were found to be more efficient for the degradation of RhB than MB under VIS light irradiation [95,97]. Complex organic molecules of antibiotics (TC, TCH, CIP, SMX, etc.) can also be efficiently photodegraded by MoS_2_-based composites. For example, the removal efficiency of TC antibiotic increased from 75% (catalyst: MoS_2_/ZnCdS, 240 min, 1 W LED lamp) [18] to 95% (catalyst: MoS_2_/CuS, 30 min, LCS-100 solar simulator) [13]. Using the same photocatalyst (MoS_2_/ZnO nanocomposite), Mohammed et al. [24] reported complete degradation of sulfamethoxazole (SMX), meloxicam (MX), and MB after 30 min, while for trimethoprim (TMP) antibiotic and malachite green (MG) dye, complete degradation was achieved after 90 min and after 120 min for the crystal violet (CV) dye. These results show that the MoS_2_/ZnO photocatalyst efficiency for tested dyes decreases in the order MB, MG, and CV, being correlated with different dyes absorption capacities. In the case of the three antibiotics, in accordance with proposed photodegradation mechanism, the degradation order is MX, SMX, and TMP. Related to the influence of light intensity/light source on the photodegradation of pollutants, it was demonstrated that almost 97% of the RhB dye was degraded by the MoS_2_/CaTiO_3_ composite (0.03 g/L) after 180 min of illumination with a 15 W LED lamp [83], while complete degradation of RhB was obtained after 24 min using a 300 W Xe lamp and the same amount of MoS_2_/Bi_4_O5Br_2_ photocatalyst in the dye solution [57].

**Figure 7:**
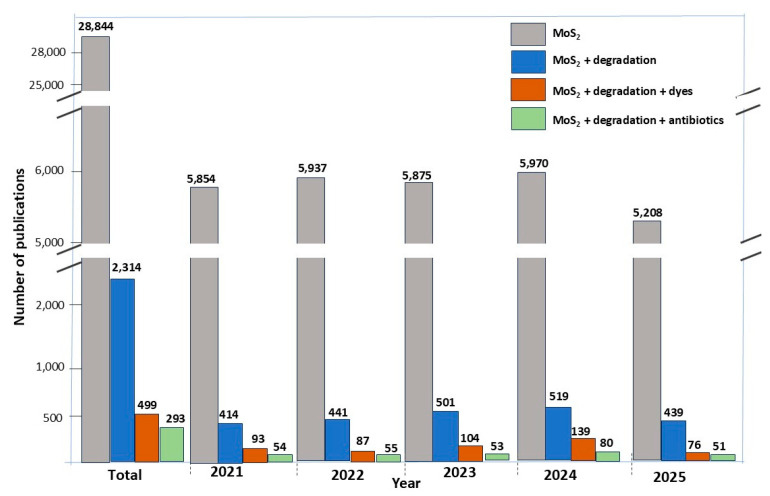
The bar chart of MoS_2_-based materials estimating research trends for dyes and antibiotics degradation in wastewater in the last five years from Web of Science (2021–2025).

According to the main Web of Science collection on MoS_2_-based materials (Figure 7), the number of publications has increased in the last five years (the estimate for 2025 is under progress), revealing that topics related to photodegradation of dyes and antibiotics are still trending in worldwide research.

### 3.1. Binary MoS_2_-Based Heterojunction Photocatalysts

#### 3.1.1. Type-I MoS_2_-Based Heterojunction Photocatalysts

Photocatalysts with MoS_2_/ZnO heterojunctions, showing different structures (0D and 2D) and improved photocatalytic activities, were synthesized via a simple or microwave-assisted hydrothermal route (Table 2) [23,24]. The MoS_2_/ZnO photocatalyst structure, obtained by a simple hydrothermal method, consists of spherically, randomly dispersed particles with an average diameter of 6.8–7.5 nm, indicating the formation of quantum dots (QDs). This type of structure (0D or QDs), more suitable for semiconductor nanomaterials due to the confinement effect of excited photons, induces different optical and electronic properties compared with those in bulk structures. Photocatalytic experiments, used for the degradation of tetracycline (TC, 20 mg/L) by irradiation with visible light (300 W halogen lamp) for 120 min, showed that the efficiency of the photocatalysts increases from 25.6% (bare ZnO), respectively 38.4% (pristine MoS_2_), to 96.5% for the MoS_2_/ZnO QDs heterostructure. The MoS_2_/ZnO QDs photocatalyst performance, with 3.8 respectively 2.5 times higher TC degradation efficiency, is attributed to the MoS_2_/ZnO heterojunction structure formed by numerous QDs with large specific surface area [23].

By coupling ZnO nanorods (NRs) with ultrathin MoS_2_ nanosheets (NSs) using a facile and green microwave-assisted hydrothermal method, MoS_2_/ZnO heterostructure nanocomposite was developed as a photocatalyst to degrade organic pollutants (antibiotics, dyes) under simulating sunlight [24]. The results showed that changes in the morphological, optical, and electronic properties of ZnO nanocrystals occur when coupled with MoS_2_’s narrower bandgap (Eg = 1.1 eV), thus improving the visible light absorption and photocatalytic activity of the nanocomposite. The faster completed photodegradation of MB (30 min), compared with those of MG (90 min) and CV (120 min), was correlated with its different chemical structure with higher adsorption capacity (about 60%) in the presence of MoS_2_/ZnO nanocomposite. In the case of the three antibiotics with low adsorption percentages (under 20%), the adsorption step had a minor effect on their photodegradation; therefore, photocatalytic activity of MoS_2_/ZnO was predominantly influenced by reactive species generated (HO•, •O_2_^−^), and the degradation order was MX, SMX, and TMP.

The developed MoS_2_/ZnO heterojunction photocatalysts with quantum dots (QDs) or 2D cluster-like structures, showed appreciable photocatalytic efficiencies (over 95%) in the degradation of antibiotics and dyes under VIS light exposure. This performance is due to the formation of the MoS_2_/ZnO heterojunction (type-I with straddling bandgaps structures), which contributed to the faster generation, separation, and transport of charge carriers in the photocatalyst.

Even though TiO_2_ is considered the most used photocatalyst for water treatment, its practical applications are still limited due to its inactivity under natural sunlight conditions [101]. To enhance the VIS light absorption response and, therefore, the photocatalytic performance, the development of Rh-photodeposited TiO_2_ nanoparticles photocatalyst was reported in [102], that where researchers selectively dehydrogenated N-heterocyclic amines (tetrahydroquinolines, tetrahydroisoquinolines, indolines, etc.), with concomitant molecular hydrogen gas generated in an inert atmosphere under VIS light illumination (λmax = 453 nm) at room temperature. As well as ZnO, TiO_2_, can form heterojunctions with other semiconductor materials (Figure 5) such as metal oxides (Cu_2_O, Co_3_O_4_), metal sulfides (CuS, CdS, MoS_2_), carbon nitride, etc.

Kumar et al. [22] reported 2D MoS_2_/TiO_2_ nanocomposite obtained through hydrothermal synthesis using various amounts of MoS_2_ (5–10 wt%), with applications in photocatalysis and rechargeable batteries. The surface morphology of the composite, consisting of TiO_2_ nanoparticles evenly distributed over the 2D MoS_2_ nanosheets with sizes ranging from 200 to 1000 nm, confirmed the formation of 2D MoS_2_/TiO_2_. The photocatalytic activity of pure TiO_2_ and MoS_2_/TiO_2_ nanocomposites, as shown in Table 2 with different MoS_2_ (5, 10 wt%), was evaluated for RhB solution (20 mg/L) degradation, under VIS light irradiation (125 W Hg-visible lamp). After 60–80 min, the dye was completely photodegraded, depending on MoS_2_%wt; it was noted that higher amounts of MoS_2_ in the nanocomposite could block the effect of TiO_2_ in the heterojunction. When pure TiO_2_ was used as photocatalyst, the dye removal was complete after 150 min, almost double the time. The increased photocatalytic efficiency in VIS light was attributed to the 2D MoS_2_ nanosheets semiconductor, which favored light absorption and efficient separation of photogenerated charge carriers through the MoS_2_/TiO_2_ heterojunction, suggesting a mechanism specific to type-I heterojunction photocatalysts. To evaluate the photocatalyst stability, recycling tests were performed, demonstrating excellent photocatalytic stability for 5%MoS_2_/TiO_2_ photocatalyst after four cycles, with a slight decrease in degradation efficiency (8%) after the fifth cycle.

The transfer of charge carriers through the interface between MoS_2_ and another semiconductor is a current methodological challenge, depending on the type of heterojunction formed and/or the structural (especially morphology), optoelectronic, and chemical properties of the heterojunction components. A strategy to improve the interface properties of heterojunction materials, therefore, charge carrier transport and separation, is interface engineering. Sasikala et al. [74] recently reported the synthesis of layered MoS_2_/TiO_2_ hybrid nanostructure with higher superior photocatalytic efficiency (94.45%) compared with pure TiO_2_ (66.6%) in CV degradation under UV light exposure for 60 min. The enhanced photocatalytic performance was attributed to composite structural coherence at the interface as a result of the crystalline nature, phase morphology, and effective heterojunction formation between MoS_2_ and TiO_2_, confirmed by XPS, TEM, HRTEM, and SAED (Selected area electron diffraction) analysis. The use of few-layer MoS_2_ nanosheets in composite structure contributed to a significant improvement in interfacial charge transfer and a reduction in defect-related recombination. Moreover, due to its narrow bandgap (1.15 eV) and superior electrical conductivity in MoS_2_/TiO_2_ heterojunction photocatalyst, MoS_2_ acted as an effective electron acceptor and transporter.

**Figure 8:**
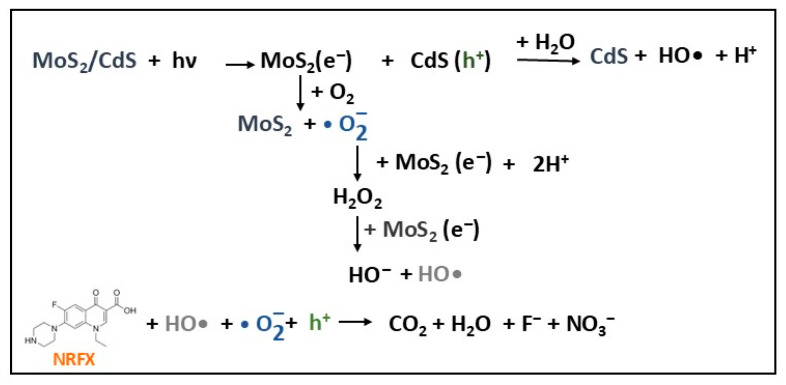
The photocatalytic degradation mechanism of NRFX antibiotic with MoS_2_/CdS photocatalyst under VIS light irradiation.

Due to the suitable positions of the CB and VB in MoS_2_ and CdS (see Figure 5), the transfer of photoinduced charges is more rapid and efficient, thus increasing the photocatalytic activity of MoS_2_/CdS heterojunction photocatalysts. In this context, a MoS_2_/CdS heterostructure photocatalyst was obtained from CdS nanorods synthesized on 2D MoS_2_ nanosheets using a solution-processable solvothermal method [30]. Both XRD and Raman analysis results confirmed the coexistence of pure CdS (preponderant) and MoS_2_ phases in composite, indicating that the MoS_2_ and CdS chemical structures were not affected by the presence of heterojunction interactions. The photocatalytic performance of MoS_2_/CdS composite was evaluated in the degradation of norfloxacin (NRFX) antibiotic in aquatic media (20 mg/L) under VIS light irradiation (Table 2). An optimal degradation efficiency of 87.5 was obtained for 10 wt% MoS_2_/CdS composite after 25 min, compared with 58% for CdS nanorods (1.5 times less) and about 10% for MoS_2_ (2.25 times less). The favorable alignment of the CB and VB levels in the heterojunction (type-I), allowed the transfer of photogenerated electrons from the CB of the CdS semiconductor to the CB of the MoS_2_ semiconductor (confirmed by PL measurements), resulting from the reduction in photogenerated charge carrier recombination, enhancing the photodegradation efficiency of the MoS_2_/CdS composite. The NRFX photodegradation reactions under visible light irradiation using MoS_2_/CdS heterojunction photocatalyst is schematically presented in Figure 8. The stability tests, including five recycling cycles, showed that unimportant changes in photocatalyst degradation efficiency occurred, resulting in a decrease of only 7.4% after the fifth cycle compared with the first cycle. This decrease was attributed to either the adsorption of NRFX remaining on the photocatalyst surface or the reduction in the MoS_2_/CdS mass after each cycle [30].

The same MoS_2_/Cd_S_ composite was investigated in photodegradation of RhB dye under VIS-light illumination [78]. It was reported that 91.9% of Rh B solution (10 mg/L) was degraded by MoS_2_/CdS photocatalyst (0.5 g/L) after 60 min of illumination. Although the degradation efficiencies of the two pollutants, NFX antibiotic and RhB dye, showed close values (difference of 4.5%), the complete degradation of the dye requires more than two times the time.

The efficiency of a photocatalyst can also be evaluated by apparent quantum yield (AQY), a key parameter that refers to the light energy harnessed in a photocatalytic process. The measured AQYs for the two photodegradation process were 0.2% (NFX) and 0.057% (RhB), with improved photocatalyst efficiency for NFX degradation. Under similar experimental conditions, the energy consumption for NFX degradation decreased 2-fold compared to CdS and 8 times compared to MoS_2_, while the energy used for RhB degradation decreased 3.15-fold (CdS nanorods) and 12.6 times (MoS_2_). These results could be correlated with a higher efficiency of the photocatalyst in RhB degradation over a longer time [30,78].

More recently, to improve photocatalytic degradation of antibiotics in wastewater, MoS_2_/Zn_0.1_Cd_0.9_S photocatalyst was obtained via a two-step hydrothermal method with soft templates, varying MoS_2_ precursors (molybdate salt and thioacetamide) concentrations [70]. The hybrid composite morphology (TEM images) consisted of uniformly loading coagulated MoS_2_ nanoflowers over smooth and well-distributed Zn_0.1_Cd_0.9_S NRs, with an average length of about 2 μm and an average diameter of 70 nm. The composite morphology significantly contributes to efficient charge carrier separation, also confirmed by PL results, by transfer of photoexcited electrons from Zn_0.1_Cd_0.9_S NRs, through the Schottky barrier, to reach the surface of MoS_2_ flower-shaped petals. Accordingly, MoS_2_/Zn_0.1_Cd_0.9_S composite degraded 99% of ofloxacin (OFX, 20 mg/L) under visible light irradiation within 2 h. Based on energy band alignments and the proposed OFX photodegradation mechanism [70], MoS_2_/Zn_0.1_Cd_0.9_S composite can be considered as a type-I heterojunction photocatalyst.

**Figure 9:**
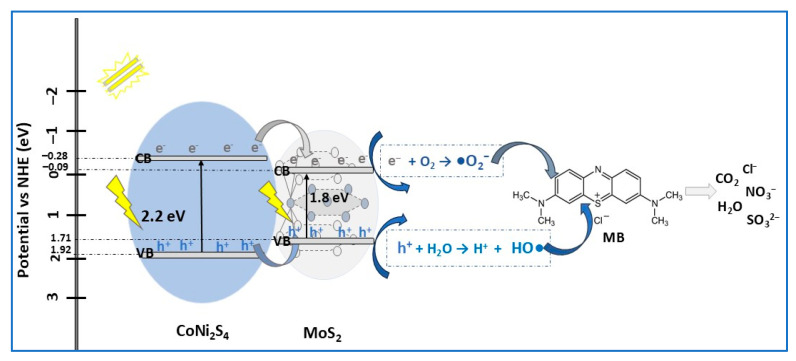
The MB photodegradation mechanism using type-I CoNi_2_S_4_/MoS_2_ photocatalyst.

Anushya and co-workers [81] obtained type-I CoNi_2_S_4_/MoS_2_ heterojunction photocatalyst via a hydrothermal method after previous Co_3_O_4_ solvothermal synthesis. The idea of heterojunction construction was to combine the catalytic stability of ternary spinel CoNi_2_S_4_ with the high light absorption of MoS_2_ nanosheets, to design an efficient photocatalyst for wastewater treatment. The estimated bandgap energies of MoS_2_, CoNi_2_S_4_, and CoNi_2_S_4_/MoS_2_ heterojunction material (with 25% wt CoNi_2_S_4_) were 1.8 eV, 2.2 eV, and 2.0 eV, respectively, confirming the apport of MoS_2_ in composite, favoring visible light absorption. As a result, under visible light exposure (500 W Xe lamp), the type-I CoNi_2_S_4_/MoS_2_ photocatalyst (0.2 g/L pollutant solution) completely degraded MB dye solution (10 m/L) within 90 min (Table 2). This photocatalytic performance is supported by the increase in the specific surface area, due to the high porosity, and by the substantial reduction of photogenerated electron–hole pair recombination (PL analysis). Therefore, efficient charge carrier separation in composite is achieved by the transfer of electrons and holes from CoNi_2_S_4_ to MoS_2_, specific to I-type heterojunction photocatalyst. Moreover, the study of different scavengers’ effects on MB photodegradation demonstrated that superoxide radicals (•O_2_^−^) are mainly responsible for dye breakdown, followed by electrons (e^−^), and then holes (h^+^), as presented in Figure 9. Based on reusability tests, after five cycles of MB degradation CoNi_2_S_4_/MoS_2_, photocatalyst efficiency decreased slightly by 7.5%, hence it could be used in applications that require long-term stability [81].

#### 3.1.2. Type-II MoS_2_-Based Heterojunction Photocatalysts

**Figure 10:**
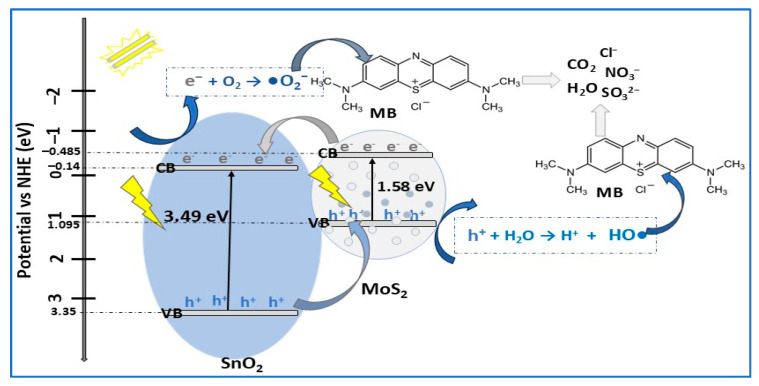
The photodegradation mechanism of MB dye in the presence of MoS_2_/SnO_2_ photocatalyst under solar simulator irradiation.

The n-type semiconductor SnO_2_, with its wide bandgap (3.6–4.1 eV), deep conduction and valence bands, high stability, and high transparency [103], demonstrated to be a promising candidate in heterojunctions with MoS_2_ for photodegradation of both organic and inorganic pollutants in water treatment. In this context, Szkoda et al. [26] prepared, using a hydrothermal method, MoS_2_/SnO_2_ composite used for photodecomposition of MB under simulated solar light illumination (Table 2). The composite surface was similar to MoS_2_ but less smooth, in which MoS_2_ microcrystals were covered with SnO_2_ NPs with an average size in the range of 5–15 nm. Using MoS_2_/SnO_2_ composite, a photocatalytic efficiency of 99.5% in MB removal was achieved after 5 min of exposure to sunlight compared to 20 min for the prepared MoS_2_. The excellent performance of binary photocatalyst was attributed to the presence of SnO_2_ in heterojunction, consequential in the significant electron–hole pair separation efficiency increasing. To investigate the role of active species (•O_2_^−^, OH•, and h^+^) in MB photodegradation, scavenger experiments were performed. It was concluded that the presence of SnO_2_ in the composite allows OH• production, therefore MoS_2_/SnO_2_ photocatalyst has higher efficiency in MB degradation. Due to the well-matched energy bands between MoS_2_ and SnO_2_ (staggered bandgaps, Figure 10), the MB photodegradation mechanism follows a type-II heterojunction scheme.

As members of the same family of two-dimensional transition metal disulfide compounds (TMDs), MoS_2_ and WS_2_ exhibit almost similar structural and physicochemical properties, as well as remarkable potential as photocatalysts. Using a chemical vapor deposition technique, MoS_2_, WS_2_, and their intermixing composites with 20% to 80% wt WS_2_ were prepared and evaluated for MB solution (5 mg/L) photodegradation under solar simulator and sunlight irradiation (Table 2) [31]. Structural analysis of the exfoliated samples obtained hexagonal 2H-MoS_2_, 2H-WS_2_, and a mixture of both crystalline phases in composite, consisting of flakes with typical shapes (triangular, hexagonal, pentagonal, etc.) and variable size, from a few hundred nanometers to a few microns. Keeping the same conditions (photocatalyst and dye concentrations, irradiation time), the photocatalytic efficiency of MoS_2_/WS_2_ composite in MB dye degradation increased from 60% when a solar simulator was used for illumination to 66.7% under direct sunlight (27 °C). These values are slightly lower than WS_2_ photodegradation efficiency (67.7%) and higher compared with MoS_2_ (43.5%), with the heterojunction formed between the two semiconductors being attributed to type-II. However, even if the composite does not stand out for its superior photocatalytic performance over 180 min, it demonstrated excellent behavior in terms of stability, degrading 97% of MB during five recycle cycles of 3 h each.

Another example of the type-II heterojunction is MoS_2_/FeOCl, synthesized by an ingenious ultrasonic method and reported to exhibit high photocatalytic degradation of organic pollutant (dyes, antibiotics) in wastewater [82]. Accordingly, iron oxychloride (FeOCl, FOC), a Fe-based heterogeneous Fenton semiconductor material, with a ternary layered structure and narrow bandgap (1.59–1.91 eV) [104] in heterojunction with 2D layered-structured MoS_2_ semiconductor, showed excellent efficiency in photo-Fenton degradation of RhB (99.5%) after 5 min and colorless antibiotic TC (90%) after 40 min irradiation in visible light. Based on the XPS (X-ray Photoelectron Spectroscopy), DRS (Diffuse Reflectance Spectroscopy), photo-Fenton, and radical trapping experimental results, due to the strong electron transfer between MoS_2_ and FOC semiconductors, the pollutant photodegradation mechanism can be ascribed to a typical type-II heterojunction scheme. The possible degradation process of RhB consists of the following steps: (1) destruction of the dye by the N-deethylation process, (2) cleavage of the chromophore, (3) ring opening by the intermediates formed in the previous step, and (4) their mineralization into CO_2_ and H_2_O.

**Figure 11:**
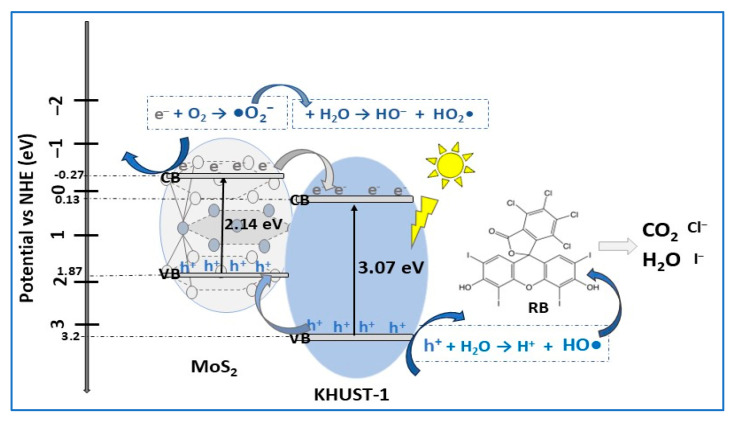
The RB dye degradation mechanism using type-II MoS_2_/HKUST-1 photocatalyst and sunlight as source of irradiation for 30 min.

Metal Organic Frameworks (MOFs) are interesting new versatile and polyfunctional materials with one- or more-dimensional porous structures resulting by metal ions/clusters coordination to organic ligands. Special properties, such as unprecedented chemical and structural tunability, large surface area, and ultrahigh porosity, make them ideal candidates for numerous applications [105]. In the MOF family, HKUST-1, containing Cu^2+^ ions coordinated to 1,3,5- benzene tricarboxylate (BTC) ligands, with a wide bandgap of 3.2 eV, has limited applicability as a photocatalyst. Designing MoS_2_ nano-sheets/Cu-MOF (MS/HK) heterojunction photocatalyst reportedly improved efficiency for dyes degradation under sunlight illumination [4]. The MS/HK composite, consisting of 3D MoS_2_ nano-sheets over the MOF octahedral structure, with an estimated bandgap energy of 1.44 eV, lower than that of HKUST-1 (Eg = 3.07 eV) and MoS_2_ (Eg = 2.14 eV), showed an excellent degradation efficiency of 96.4% in Rose Bengal (RB) dye removal under sunlight exposure for 30 min. High photodegradation efficiencies, varying from 63.6% for Naphtol Green B (NGB) to 82.1% for Methyl Orange (MO), were also reported for other dyes (Table 2). The photodegradation of dyes was explained by association with wide bandgap HKUST-1 with a narrow bandgap semiconductor (MoS_2_), in a type-II heterojunction that enhanced visible light absorption, with effective separation and transport of photogenerated charge carriers. The proposed photodegradation type-II scheme mechanism (Figure 11) was supported by photoluminescence and radical trapping experiments.

**Figure 12:**
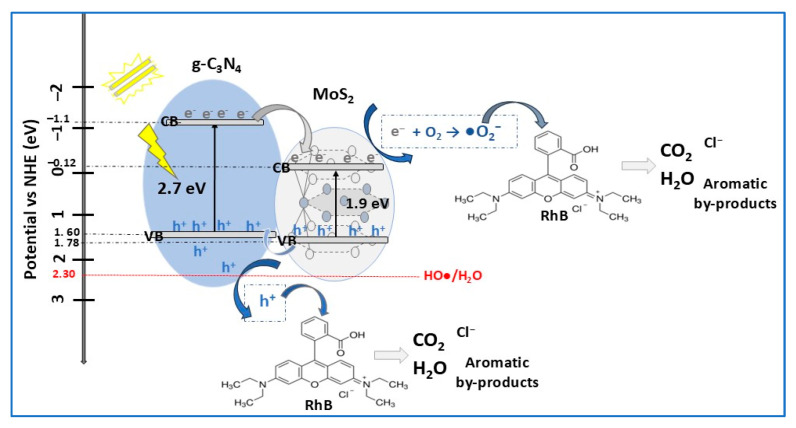
The RB dye degradation mechanism using g-C_3_N_4_/MoS_2_ photocatalyst.

Graphitic carbon nitride (g-C_3_N_4_) is a relatively new non-metallic semiconductor material with two-dimensional (2D) g-C_3_N_4_ nanosheets and stable physical and chemical properties, moderate bandgap (2.6–2.7 eV), and sensitive visible light response [40,106]. However, the pristine g-C_3_N_4_ photocatalyst encountered some drawbacks related to the photogenerated electrons’ tendency to combine with holes through the complexation process. To promote charge separation, g-C_3_N_4_/MoS_2_ heterojunction nanomaterial was prepared by an impregnation calcination method [40]. Depending on calcination temperature, the nanocomposites adopted different morphologies. The uniform-thickness multilayer lamellar nanosheets (NS) morphology with predominant g-C_3_N_4_ nanosheets were obtained at 550 °C. At lower calcination temperatures, the morphology was partly flaky due to agglomeration phenomena, while at higher temperatures a fractured multilayer lamellar structure was observed. To investigate photocatalytic performance, experimental tests were carried out using g-C_3_N_4_ NS, MoS_2_ NS, and g-C_3_N_4_/MoS_2_ composite for degradation of RhB solution (10 mg/L) under VIS light illumination for 90 min. The degradation efficiencies were close, with values of 91%, 91.5%, and 99.4%, respectively. The photodegradation rate of the nanocomposite was found to be 2.1 times more that of g-C_3_N_4_ NS, indicating the stronger capacity of binary composite to degrade RhB. The suitable band energy structure in g-C_3_N_4_/MoS_2_ heterojunction (type-II) enhanced the photocatalytic activity of composite by improving the transmission and inhibiting the recombination of photogenerated carriers. In the photocatalytic mechanism for RhB degradation by g-C_3_N_4_/MoS_2_ composite, depicted in Figure 12, h^+^ and •O_2_^−^ played the main roles in RhB dye decomposition through a redox process [40].

#### 3.1.3. Z-Scheme MoS_2_-Based Heterojunction Photocatalysts

Luo et al. [83] used a simple template-free method to construct a novel Z-scheme MoS_2_/CaTiO_3_ (CTO) heterostructure with hydrothermally synthesized MoS_2_ nanoflower deposited on the surface of rectangular CaTiO_3_. Due to its strong ability to reduce/oxidize photogenerated charge carriers, perovskite (CTO) uses are limited to only under UV light illumination. An efficient way to extent its application to sunlight conditions is coupling with MoS_2_ semiconductor in a heterojunction structure.

The well-deposited MoS_2_ nanoflowers on the surface of CTO material caused a bandgap reduction from 3.15 eV (CTO) to 3.06 eV, considerably improving its light energy absorption properties and photodegradation efficiency. Compared with bare CTO (19% efficiency), the MoS_2_/CTO heterojunction photocatalyst showed significantly enhanced photocatalytic performance (97% efficiency) in RhB dye degradation in water under VIS light irradiation over 180 min. The improved photocatalytic performance was attributed to both the strong interactions between the MoS_2_ nanoflowers and rectangular CTO, but also to direct Z-type heterojunction formation, which allows an efficient and versatile pathway to accelerate photogenerated charge carrier separation and direct their transport through the composite. Moreover, the MoS_2_/CTO photocatalyst exhibited significant stability, the photocatalytic efficiency decreasing only 4% after five cycles of RhB photodegradation. In the Z-scheme mechanism, electrons transported from the VB to the CB reduced the dissolved O_2_ in water to •O_2_^−^, which breaks down the complex RhB molecule into smaller particles due to the CB potential position of CTO (−0.69 eV), which is more negative than the CB potential of O_2_/•O_2_^−^ (−0.33 eV). Simultaneously, the holes in VB of the two semiconductors contributed both to the degradation of RhB by oxidation but also to the decomposition of H_2_O molecules into highly oxidative active hydroxyl radicals (•OH).

**Figure 13:**
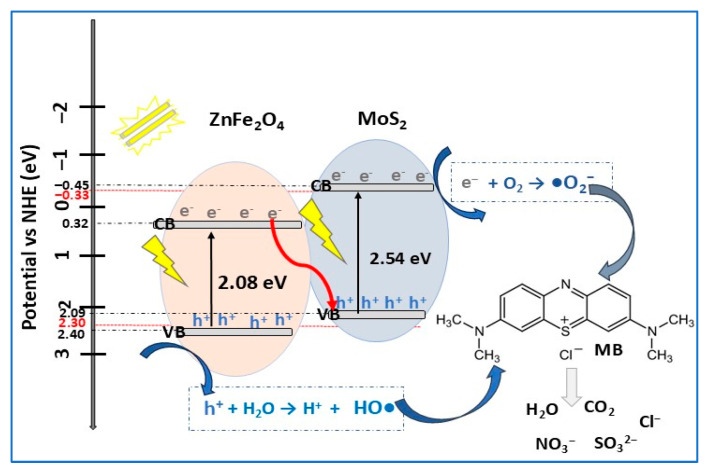
Direct Z-scheme mechanism for MB dye photodegradation with MoS_2_/ZnFe_2_O_4_ photocatalyst under VIS light illumination for 150 min.

ZnFe_2_O_4_ or ranklinite (zinc ferrite) is a versatile cubic spinel ferrite magnetic semiconductor (Eg = 1.7–3.3 eV) with applications in photocatalysis for wastewater treatment, energy storage (batteries, supercapacitors), and biomedicine. In contrast to its advantages, such as its low cost, environmental friendliness, and photochemical stability, several disadvantages have been reported for ZnFe_2_O_4_, including its high aggregation tendency due to its high surface energy [107]. This inconvenience can be reduced or even eliminated by Z-scheme heterojunction construction with a suitable semiconductor. Recently, a direct Z-heterojunction MoS_2_/ZnFe_2_O_4_ photocatalyst with a disk-like structure (MoS_2_) along with ZnFe_2_O_4_ spherical nanoparticles (10–20 nm) and bandgap energies in the range of 2.54–4.01 eV (depending on ZnFe_2_O_4_ amount in composite) was synthesized via a hydrothermal method [80]. The photocatalytic experiments (Table 2) showed that 92.3% of MB was degraded in about 150 min compared with 32.1% in the absence of photocatalyst (almost three times more). The high photocatalytic activity of MoS_2_/ZnFe_2_O_4_ composite (with 25% ZnFe_2_O_4_) was due to the enhanced light absorption and charge separation induced by direct the Z-scheme heterojunction MoS_2_/ ZnFe_2_O_4_. It was reported that not only the photocatalyst composition and dosage influenced its photocatalytic performance, but also dye solution concentration and pH. Thus, by increasing the concentration of the MB solution from 5 mg/L to 20 mg/L, the photodegradation efficiency of the composite decreased, due to the photocatalytic active sites surrounded by more particles, resulting in electron/hole pair generation decreasing and light obstruction at the photocatalyst surface. To elucidate the MB degradation mechanism, scavenger experiments were carried out. The results highlighted that photogenerated reactive hydroxyl radicals (OH•) were the main active species in MB degradation, and the direct Z-scheme mechanism, presented in Figure 13, was proposed.

#### 3.1.4. S-Scheme MoS_2_-Based Heterojunction Photocatalysts

Tuning the bandgap in S-scheme heterojunctions plays an essential role in broadening the absorption of the solar spectrum, preventing the recombination of electron–hole pairs and improving their redox abilities. Also, efficient solar energy conversion occurs on photocatalysts with large surface areas and extended active centers [108].

**Figure 14:**
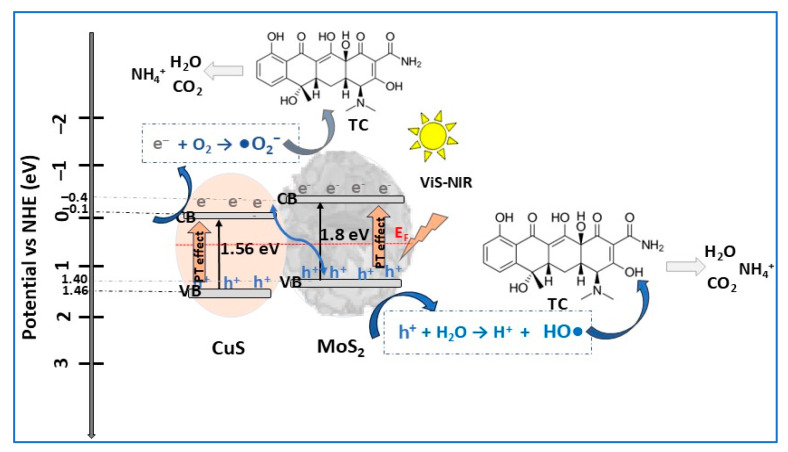
S-scheme mechanism for TC antibiotic photodegradation with CuS/MoS_2_ photocatalyst under VIS–NIR light irradiation and ultrasonic vibration for 30 min.

By growing MoS_2_ nanosheets on the surface of CuS microspheres via hydrothermal methodology, Tran et al. [13] developed a performant CuS/MoS_2_ p–n heterojunction photocatalyst, integrating piezoelectric and photothermal enhancement effects. In composite, numerous MoS_2_ nanosheets with flower-like morphology are uniformly disposed on the surface of CuS microspheres 1–2 μm in diameter. The number of MoS_2_ nanosheets on the surface of CuS microspheres increased with increasing amounts of MoS_2_ (Mo/Cu mass) in composite. At a higher Mo/Cu mass ratio (100:1), the photocatalytic activity of the composite substantially decreased due to MoS_2_ nanosheets aggregation and dispersion on the surface of the CuS microspheres, blocking heterojunction formation. The optimized CuS/MoS_2_ photocatalyst composition was that with a 50:1 Mo/Cu mass ratio. The photocatalytic activities of CuS, MoS_2_, and CuS/MoS_2_ composite were evaluated by studying the degradation of TC solutions (20 mg/L) under VIS irradiation, VIS–NIR irradiation, and ultrasonication for 30 min. As expected, the CuS/MoS_2_ heterostructure showed significantly better photocatalytic efficiency, increasing from 57% (VIS irradiation) to 95% (VIS–NIR irradiation and ultrasonication) compared with CuS (30% to 45%, respectively) or MoS_2_ (39% to 62%, respectively), as a consequence of the synergistic effect from the combination of piezoelectricity and photothermal conversion induced by p–n heterojunctions. The piezo- and photothermal-assisted photocatalytic mechanism of CuS/MoS_2_ composite under VIS–NIR irradiation and ultrasonic vibration is displayed in Figure 14. The p–n heterojunction energy band diagram, with CuS and MoS_2_ bandgaps of 1.56 eV and 1.80 eV, respectively, could be associated with the S-scheme type, in which the separation of photogenerated carriers is enhanced by the transition of photoexcited electrons and holes between the two semiconductors. This enhancement was attributed to the combination of the two effects, the piezoelectric effect of MoS_2_ nanosheets and photothermal (PT) conversion of both semiconductors in the CuS/MoS_2_ heterojunction photocatalyst [13].

An efficient strategy to design performant BW/IEM p–n heterojunction photocatalysts was reported to be the coupling of an expanded MoS_2_ (IEM) interlayer with Bi_2_WO_6_ (BW), using a simple two-step hydrothermal method [84]. During the synthesis, the BW spherical nanoparticles (∼56 nm diameter) were deposited on the surface of random distributed MoS_2_ nanoplate-like structures, confirming BW/IEM heterostructure formation. Even if the surface morphology of BW/IEM composites is mainly similar to that of IEM, due to the intercalation of BW NPs (2.9 eV) in IEM (2.2 eV) structure, the evaluated bandgap energy (2.7–2.9 eV) was closer to that of BW. The increase in bandgap energy did not affect the photocatalytic activity of the composite, which was demonstrated by the 97% photocatalytic efficiency (4% Bi_2_WO_6_/MoS_2_) in MB dye degrading under low power 1 W LED white light illumination for 60 min. According to XPS results, the observed binding energy shift in Mo 3d, S 2p, and Bi 4f peaks, indicating the presence of a strong interaction between MoS_2_ and Bi_2_WO_6_; this suggests that at their interface a charge transfer occurred, corresponding to a p–n heterojunction. Reusability tests (five MB photodegradation cycles) demonstrated excellent performance in terms of long-term stability of the 4% BW/IEM composite, with a photodegradation decrease of about 1%, confirming the formation of a strong and efficient p–n heterojunction [84].

**Figure 15:**
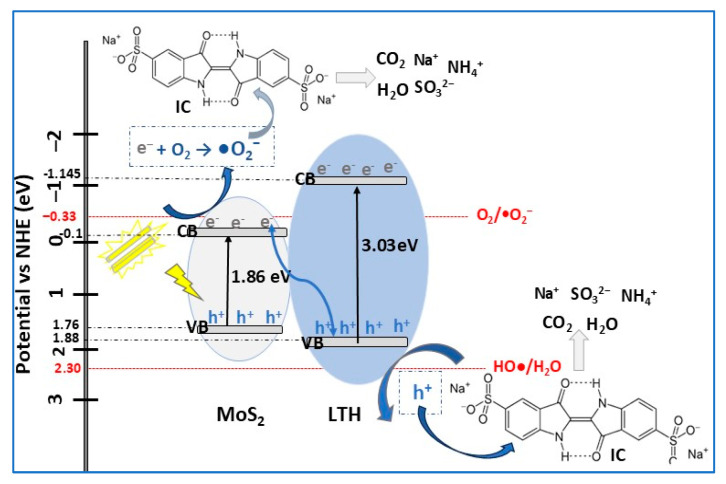
S-scheme mechanism for IC dye photodegradation with MoS_2_/NiAlFe-LTH photocatalyst under VIS light illumination for 200 min.

An ecological challenge in wastewater treatment by photocatalysis is the removal of indigo carmine (IC) dye, which, when discharged in large quantities, greatly increases the water pH (11–14). To eliminate this impediment, MoS_2_/LTH photocatalysts consisting of n-type NiAlFe-layered triple hydroxide (LTH) loaded with various ratios (1, 1.5 and 2.5) of p-type MoS_2_ were prepared through an in situ hydrothermal strategy [9]. The MoS_2_/LTH composites showed a well-defined plate-like morphology consisting of 2D layered NPs, structure that provides excellent routes for electron diffusion, facilitating photogenerated electron transfer and migration in the composites. To obtain more in-depth information about the MoS_2_/LTH surface properties, BET surface area analysis was performed. The results indicated mesoporous structures with pore sizes in the 7.60–18.98 nm range and specific surface areas (S_BET_ = 19.95–92.21 m^2^/g) lower than that of LTH (S_BET_ = 130.02 m^2^/g), depending on the amount of MoS_2_ in the composite. The addition of MoS_2_ into LTH influenced also the direct bandgap energy, which decreased from 3.03 eV (LTH) to 2.59–2.74 eV for MoS_2_/LTH heterojunction composites. The photocatalytic activity of MoS_2_/LTH materials were tested in IC dye degradation exposed to VIS light for 200 min (Table 2). The optimized photocatalyst (LM1, LTH:MoS_2_ = 1:1) degraded 100% of IC dye in high alkaline pH conditions, with a degradation rate 15 times higher compared with that of pristine LTH. This enhanced photocatalytic activity was attributed to the synergistic effect between the two semiconductors, MoS_2_ and NiAlFe-LTH, and p–n heterojunction formation. The IC dye photodegradation schematic proposed by the authors is illustrated in Figure 15.

The charge carrier transfer between LTH and MoS_2_, specific to the S-scheme mechanism, consists of the migration of photogenerated electrons from the CB of MoS_2_ (p-type semiconductor) to the CB of NiAlFe-LTH (n-type semiconductor), and photogenerated holes from the VB of NiAlFe-LTH to the VB of MoS_2_, due to the suitable energy band alignment and built-in electric field at the heterojunction interface. Electrons from the CB of p-type semiconductor directly reduced dissolved O_2_ to generate •O_2^−^_ radicals (MoS_2_ E_CB_ < E^0^_O2/•O2_^−^), which react with IC dye molecules to form non-polluting species (CO_2_, H_2_O, and inorganic ions). On the other hand, the holes on the NiAlFe-LTH could not react with H_2_O to generate •OH radicals because E^0^_•OH/H2O_ (+2.3 eV) is more positive than the LTH E_VB_; as a result, these holes react with IC dye directly, and intermediate products are obtained [9]. Recycling tests showed that after four cycles of IC dye photodegradation, the photocatalytic activity decreased by ~7% due to the adsorption of IC dye on the photocatalyst surface. It can be concluded that both the photocatalytic and photostability performances substantiate MoS_2_/NiAlFe-LTH photocatalyst as being effective for the treatment of dye-laden wastewater.

### 3.2. Ternary MoS_2_-Based Heterojunction Photocatalysts

Carbon-based materials, such as graphene, graphene oxide (GO), carbon nanotubes (CNTs), carbon quantum dots (CQDs), carbon nanofibers (CNFs), and graphitic carbon nitride (g-C_3_N_4_), with a large specific surface area, excellent physicochemical stability, and electrical conductivity, have been reported as efficient supports for photocatalysts by integration into Z- or S-type heterojunctions with semiconductor(s) [109].

The facile, flexible, and economical carbon nanotubes (CNTs) bridged to MoS_2_/ZnO nanohybrid photocatalysts (MZCs) were obtained by hydrothermal synthesis, using different mass ratios of CNTs: 1, 10, 15, 20, and 25 mg [36]. It was reported that MZC nanohybrids, with spherical or quasi-spherical nanoparticle (20–50 nm diameter) morphology and exhibiting bandgap energies in the range 2.61–2.74 eV, effectively absorbed visible light. As a result, the photocatalytic efficiencies in Tetracycline (TC) aqueous solutions (10 mg/L) degradation under VIS light exposure for 60 min varied for MZC nanohybrids from 75.3% (MZC-1) to 95.6% (MZC-25); meanwhile, for bare MoS_2_, ZnO, and CNTs, the obtained values were lower: 26.2%, 33.8%, and 24.7%, respectively. In order to study the possible photodegradation mechanism of the Z-scheme MZC nanohybrids, the TC degradation intermediates were analyzed, and the proposed TC photodegradation pathway included (1) hydroxylation, (2) dehydrogenation of intermediary compounds resulting from stage (1), (3) oxidation of intermediates to compose product, (4) dehydration, (5) deamination when intermediates are decomposed to aromatic compound, and (6) ring opening reactions when intermediates are transformed into small-molecular inorganic species (H_2_O, CO_2_, and NH^4+^). The results demonstrated the importance of ternary Z-scheme heterojunction formation, with carbon nanotubes (CNTs) as electron bridges between MoS_2_ and ZnO semiconductors. In addition, the CNT bridges allowed the introduction of carboxyl and hydroxyl functional groups in the Z-scheme heterojunction mechanism, facilitating the adsorption of organic compounds on the photocatalyst surface, thus improving its photocatalytic performance. According to energy-band alignments of MoS_2_ and ZnO semiconductors, in the Z-scheme mechanism, photoexcited electrons on the ZnO CB could not reduce O_2_ to •O_2_^−^; as well, holes from the VB of MoS_2_ could not oxidize H_2_O molecules to HO• radicals. However, photo-induced electrons from the MoS_2_ CB are removed from the MoS_2_ CB through CNT bridges to the ZnO CB where they react with O_2_ to form superactive •O_2_^−^ radicals, which further decompose TC pollutant. At the same time, the holes in the MoS_2_ VB are transferred over the CNTs to the ZnO VB and react with H_2_O molecules, resulting in HO• radicals that also decompose TC molecules into CO_2_, H_2_O, and intermediate compounds. Thus, MZC heterostructure photocatalysts can exhibit excellent charge carrier transport capacity, significantly reducing the electron–hole pair recombination rate. Moreover, compared with binary MoS_2_/ZnO composites [23], MoS_2_/ZnO/CNTs ternary heterojunction photocatalyst showed enhanced photocatalytic performance (stability and efficiency) in TC degradation under VIS light irradiation due to the construction of all-solid-state Z-scheme heterojunction with CNTs as mediator.

**Figure 16:**
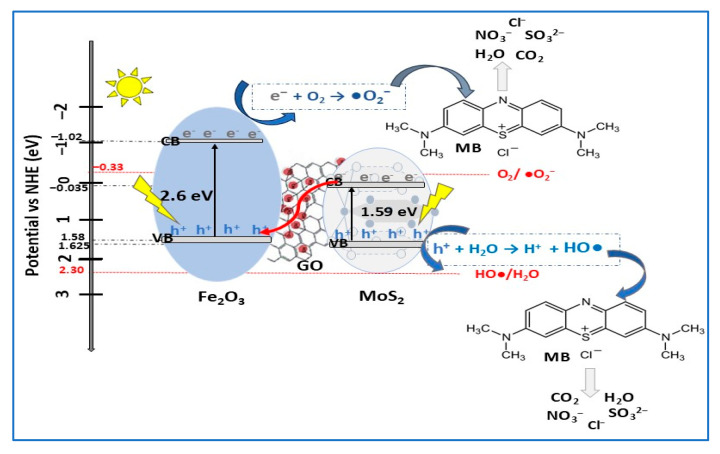
Z-scheme mechanism for MB dye photodegradation using MoS_2_/Fe_2_O_3_/GO composite photocatalyst and simulated solar radiation.

Recently, Samarasinghe and co-workers [37] obtained MoS_2_/Fe_2_O_3_/GO (MFG) heterojunction composites as highly efficient, stable, and reusable photocatalysts in textile wastewater treatment. The ternary MoS_2_-based composites, with an optimal mass ratio of 2:1:1, were synthesized through ball milling and ultrasonication techniques. The heterojunction interface structure (Fe_2_O_3_ spheres uniformly distributed on the MoS_2_ sheets and GO matrix), bandgap energy of 1.9 eV (intermediary between those of MoS_2_ and Fe_2_O_3_), and two-fold increase in specific surface BET area compared with MoS_2_ significantly improved MFG composite photocatalytic activity in dyes degradation. As expected, the MZC heterojunction composite exhibited a remarkable photocatalytic efficiency of 97.90% in the degradation of MB within 3 h under simulated solar irradiation, while under natural sunlight the efficiency decreased almost 10%. This increased efficiency of MB dye degradation was correlated with the development of the solid-all-state Z-scheme heterojunction, which favored the separation of charge carriers, eliminating the recombination of photogenerated electron–hole pairs following electron transfer from MoS_2_ to Fe_2_O_3_ via the graphene oxide (GO) sheets bridge. The MB photodegradation Z-scheme mechanism is depicted in Figure 16. In addition to its remarkable photocatalytic activity, stability, and reusability, the performance of the MoS_2_/Fe_2_O_3_/GO photocatalyst in natural sunlight makes practical application possible on a large industrial scale, under real environmental conditions, thus reducing energy consumption from using artificial light sources [37].

**Figure 17:**
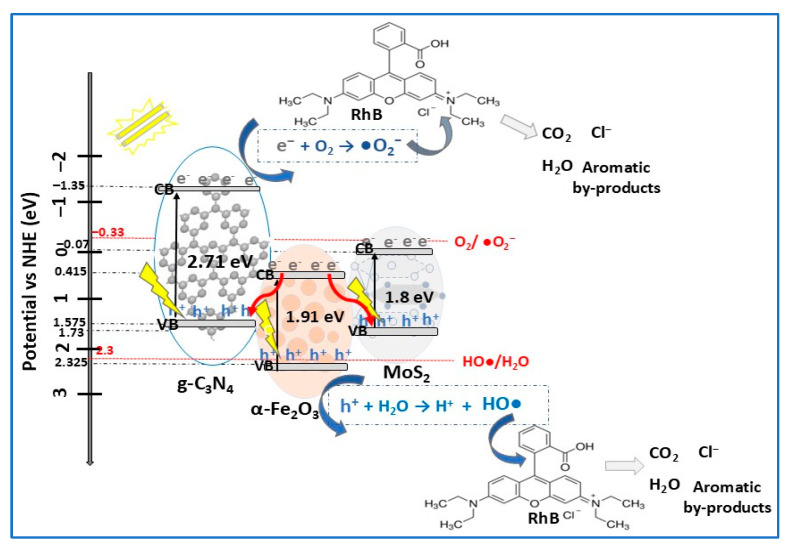
Z-scheme mechanism for RhB dye photodegradation with ternary g-C_3_N_4_/α-Fe_2_O_3_/MoS_2_ composite photocatalyst.

Compared to the binary composite photocatalyst g-C_3_N_4_/MoS_2_, which degraded 99.4% of RhB dye in 90 min of exposure to visible light [40], the ternary nanocomposite g-C_3_N_4_/α-Fe_2_O_3_/MoS_2_ (GFMO) obtained via calcination followed by hydrothermal synthesis achieved a RhB photodegradation efficiency of 95.6% in 80 min [95]. Even though the morphologies (NS MoS_2_ and NS g-C_3_N_4_) and efficiencies are quite close, the presence of α-Fe_2_O_3_ semiconductor (co-catalyst) in the ternary composite caused the modification of the heterojunction interface by coupling g-C_3_N_4_ with MoS_2_/α-Fe_2_O_3_. In this GFMO heterojunction structure, α-Fe_2_O_3_ catalyst acted as an oxidation center that generates multiple reactive sites, facilitating visible light absorption, efficient charge carrier separation and synergistic Z-Scheme heterojunction photoreactions. The scavenging tests showed that •O^2−^ and h+ are the most reactive species with important roles in RhB dye photocatalytic degradation via traditional Z-scheme mechanisms (Figure 17).

**Figure 18:**
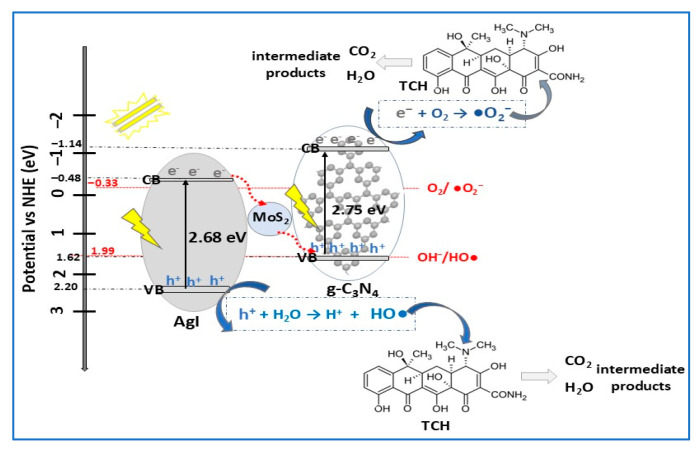
TCH antibiotic photodegradation all-solid-state Z-scheme mechanism in the presence of ternary g-C_3_N_4_/MoS_2_/AgI photocatalyst.

An interesting example of efficient Z-scheme heterojunction photocatalyst, with MoS_2_ QDs as charge transfer mediator, was tested for tetracycline hydrochloride (TCH) degradation under VIS light irradiation [94]. MoS_2_ QDs decorated g-C_3_N_4_/AgI heterostructure composite, with Eg = 2.7 eV, and degraded 82.8% of TCH in 50 min, showing a higher photocatalytic activity than bare g-C_3_N_4_ (48.1%) and binary g-C_3_N_4_/MoS_2_ composite (75%). Based on PL, Mott–Schottky, and scavenging analysis results, the TCH photodegradation mechanism proposed for ternary g-C_3_N_4_/MoS_2_/AgI composite is presented in Figure 18. In this all-solid-state Z-scheme mechanism, the photogenerated charge carriers, e.g., electrons on the CB of AgI and holes from the VB of g-C_3_N_4_, are recombined through MoS_2_ QD electron mediators. The efficiency of charge carrier separation and migration in the g-C_3_N_4_/MoS_2_/AgI composite was significantly improved due to the Z-pattern heterojunction, which favors the increased production of predominant superactive radicals •O_2_^−^ and •OH during the photocatalytic process.

**Figure 19:**
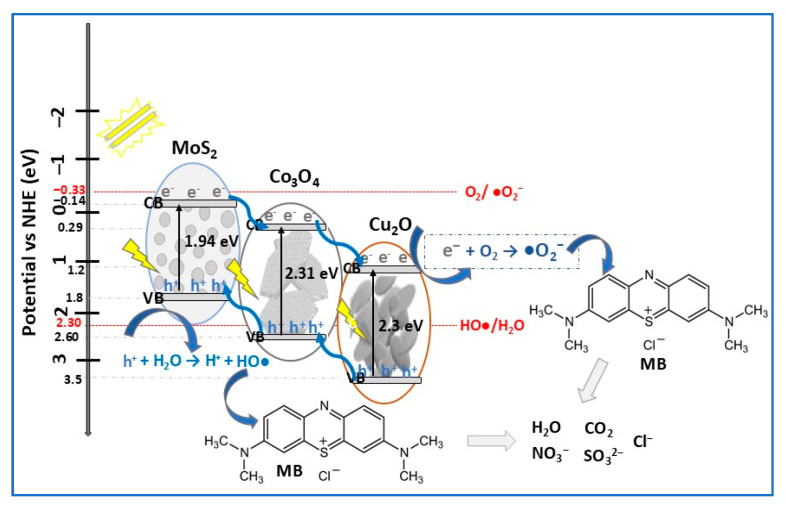
S-scheme mechanism for MB dye photodegradation with ternary MoS_2_/Co_3_O_4_/Cu_2_O composite photocatalyst under VIS light irradiation for 90 min.

Among all transition metal oxides (TMO), tricobalt tetroxide (Co_3_O_4_) and copper (I) oxide (Cu_2_O) are both p-type semiconductors, with bandgap energies of 1.29–5 eV [110] and 2.17 eV [111], respectively, and promising properties including low cost, nontoxicity (Cu_2_O) and less toxicity in low amounts (Co_3_O_4_), high stability (excepting acidic environments for Co_3_O_4_), and visible light absorption ability. However, in practical large-scale photocatalysis applications, pristine Co_3_O_4_ and Cu_2_O semiconductors have still limitations due to the rapid recombination process of photogenerated charge carriers, thus poor photocatalytic activity. To enhance their performance by morphological tailoring and semiconductor heterojunction construction, recent research focused on MoS_2_/Co_3_O_4_/Cu_2_O nanocomposite prepared by facile sonication-assisted hydrothermal methods [97]. Morphology studies (SEM and TEM) revealed that the self-assembled MoS_2_/Co_3_O_4_/Cu_2_O nanocomposite is a mix of nanosheets (Co_3_O_4_), nanoflakes (Cu_2_O), and nanoparticles (MoS_2_) with different sizes. In addition, PL experiments confirmed that both Co_3_O_4_ and Cu_2_O nanostructures were formed on the surface of MoS_2_ NPs, specific to this ternary nanocomposite, in which Cu_2_O acts as a co-catalyst for the MoS_2_/Co_3_O_4_ p–n heterojunction photocatalyst. To evaluate the photocatalytic activity, prepared photocatalysts (MoS_2_, Co3O4, Cu_2_O, MoS_2_/Co_3_O_4_, MoS_2_/Co_3_O_4_/Cu_2_O) were tested in degradation of MB and RhB dyes under UV–VIS light irradiation. The ternary nanocomposite showed the highest photocatalytic degradation efficiency, increasing from 43% (MoS_2_) to 91% for MB (100 min of light irradiation), and from 47% (MoS_2_) to 92% for RhB, after 90 min. Based on morphological, bandgap, PL analysis, and scavenging studies, the S-Scheme photocatalytic mechanism (Figure 19) was proposed for organic dyes degradation using MoS_2_/Co_3_O_4_/Cu_2_O heterojunction photocatalyst. Ternary heterojunction structures have a complex system of photocatalytic reactions than simple and binary systems, the third component playing an important role (mediator) in charge carrier transfer during reactions. In MoS_2_/Co_3_O_4_/Cu_2_O nanocomposite, Cu_2_O acted as an excellent n-type co-catalyst (mediator) for the p–n heterojunction (MoS_2_/Co_3_O_4_) naturally formed, upon light irradiation. To promote higher oxidation holes, the electron transfer mechanism follows the route from the MoS_2_ CB to the Cu_2_O CB, while to higher reduction electrons stimulation the holes are transferred in the opposite way, from the Cu_2_O VB to the MoS_2_ VB. The photogenerated electrons and holes undergo oxidation–reduction reactions to produce a higher number of active •OH and •O_2_^–^ radicals, which decompose organic dyes molecules. Based on this innovative S-scheme mechanism, in addition to efficient charge transfer, electron–hole pairs are easily induced and then separated, reducing their further recombination, therefore enhancing ternary photocatalyst performance. Consequently, MoS_2_/Co_3_O_4_/Cu_2_O nanocomposite, with its very good stability, low cost, and high efficiency in persistent organic pollutants (dyes) removal, without generating secondary harmful compounds, represents a feasible choice for sustainable wastewater treatment.

Based on the above discussions, a potential critical evaluation, including the advantages and limitations of MoS_2_-based photocatalysts, according to heterojunction type, is presented in Table 3. The advantages of each type of heterojunction were considered, as well as their limitations in terms of the pollutant photocatalytic degradation mechanism and the studied photocatalytic system (catalyst, pollutant, working conditions, etc.). Some examples are also mentioned.

## 4. Current Challenges and Future Research

Current challenges and research on MoS_2_-based photocatalysts are generally related to charge carrier recombination, the number of active sites, low conductivity for the 2H-phase, low stability for conductive 1T-phase, scalable advanced material design, etc. [49].

The photocatalytic performance of layered MoS_2_ is influenced by its crystalline phase composition, which could be modified by expanding the interlayer spacing [112]. Among current strategies applied, such as heteroatoms doping, defect engineering, etc., phase engineering has been considered an innovative approach to transform or combine MoS_2_ (1T, 2H) to obtain enhanced photocatalytic properties. It was reported [113] that MoS_2_ nanosheets combining 1T/2H phase showed excellent efficiency (95%) in the photodegradation of MO compared to 2H phase MoS_2_ (12%) due to 1T sites insertion into 2H layers, increasing MoS_2_ conductivity and photocatalytic activity. To improve the co-catalytic performance of MoS_2_ for Fe^3+^-mediated Fenton-like technology, Xiao and colleagues [114] prepared a C2-MoS_2_/Fe^3+^/PMS (peroxymonosulfate) system by carbon doping the S-defective 1T/2H mixed phase MoS_2_. The system demonstrated excellent degradation efficiency (approximately 100% in 10 min) of the antibiotic sulfadiazine (SDZ), demonstrating promising prospects in the removal of antibiotic pollutants in wastewater. Future research should consider more advanced synthesis techniques to develop 1T/2H MoS_2_ hybrid structures with high stability and (photo)catalytic activity, as well as for more precise morphological control to increase surface area and the number of active sites. Further research should be directed towards more advanced synthesis techniques to design and develop 1T/2H MoS_2_ hybrid structures that, partially or totally, alleviate the stability and catalytic activity issues.

In the last few years, advanced membrane-based technology has proven to be a viable and powerful tool for producing clean water, due to its convenience and energy efficiency in wastewater treatment. Recent advances in MoS_2_-based membranes included the design, fabrication, and application in wastewater treatment of various types of membranes, i.e., nanoporous, layer-stacked, composite membranes [115]. To overcome limitations related to the reusability and fouling of the membranes used in textile wastewater treatment, PVDF (polyvinylidene fluoride)/TiO_2_-MoS_2_ nanocomposite membrane was recently developed [116]. The new nanocomposite membrane showed good filtration performance (90–95%) for Reactive Yellow (RY), Acid red (AR), and Navy XF (NXF) colorants. These results were correlated with filtration mechanisms, which combine photocatalytic degradation of organic contaminants attached to the membrane surface with its self-cleaning properties. Further research must be concentrated on improving performance, stability, and cost-effectiveness of MoS_2_-based membranes for expanded applications such as wastewater treatment, water desalination, water and air purification, etc.

Another challenge related to MoS_2_-based photocatalysts is their long-term stability, with a significant influence on the economic feasibility of the photodegradation process [117]. Therefore, future studies on the reuse and photostability of MoS_2_-based photocatalysts are obviously needed to evaluate their ability to regenerate more than four or five times without a significant decrease in their photodegradation efficiency of organic pollutants.

Another challenge addressed to pristine MoS_2_ and MoS_2_ heterojunction photocatalysts is related to their photocatalytic activity, which has been extensively studied mainly on model systems and less on real wastewaters that contain a complex mix of organic and inorganic contaminants. Moreover, due to the limited available data about factors influencing the performance of MoS_2_-based photocatalysts, future research on pollutant photodegradation should be performed in extended experimental conditions, including working temperature and pH, photocatalyst dosage, initial concentration(s) of contaminant(s), the type and intensity of irradiation sources, the distance from the light source, etc.

## 5. Conclusions

As discussed in this review, there have been significant advances and improvements in the photocatalytic performance of MoS_2_-based photocatalysts to remove persistent organic pollutants (dyes, pharmaceutical active compounds, pesticides, phenol, and derivates) in wastewater treatment. To achieve higher efficiency in pollutant photodegradation, even under natural sunlight irradiation conditions, recent research has focused on tailoring MoS_2_-based photocatalysts properties using different strategies, such as morphology engineering, metal doping (Ag, Au, Sn), and heterojunction development (type I, type II, Z- and S-schemes), which has resulted in improved photocatalytic performance. Therefore, in this review, a particular importance has been placed on designing and developing stable MoS_2_ heterojunction photocatalysts with enhanced performance compared with pristine MoS_2_ photocatalyst. Even though certain photocatalysts have demonstrated complete or nearly complete degradation efficiency for both dyes (90–100%) and antibiotics (80–100%) under VIS light irradiation, the technology used remains restricted to laboratory-scale research. Thus, their limited large-scale production and commercialization under realistic environmental conditions remains an open issue that needs to be explored. Further research should focus on exploring the immense potential of MoS_2_ and identifying new high-performance MoS_2_-based heterojunction photocatalysts suitable for the degradation of persistent organic pollutants in wastewater, with possible extension to other applications.

## Figures and Tables

**Table 1:** The morphological, optical, and photocatalytic properties of MoS_2_ and metal (Ag, Sn)-doped MoS_2_ photocatalysts.

Photocatalyst Morphology	Eg eV	S_BET_ m^2^/g	Dye	λ_max_ nm	η* %	t min	Ref.
MoS_2_ 3D flower-like hemispheres (d ≈ 2.5 μm)	2.05	-	RhB MB	554 665	81.5 91.6	120	[1]
MoS_2_ nanoflowers (d ≈ 100 nm)	2.2	-	RhB	554	39.9	120	[34]
MoS_2_ layered nanostructures	-	50	MB CV	665 590	83 (UV) 73 (sun) 71 (UV) 57 (sun)	90	[2]
MoS_2_ NPs (d ≈ 70 nm) MoS_2_ (M-UREA) NRs (d ≈ 25 nm)	2 1.96	45 29	industrial (leather) wastewater	-	45 59	180	[5]
MoS_2_ irregular microspheres (d ≈ 500 nm)	-	-	TBC	221	95	720	[7]
MoS_2_ nanoflakes (d ≈ 13 nm) MoS_2_ biosynthesized nanoflakes (d ≈ 4–6 nm)	2.37 2.03	79 121–134	OTC	376	96 98–99	120	[35]
MoS_2_ nanopetals Ag-MoS_2_ nanopetals (reduced d and thickness)	2.35 1.55	-	MB	665	40 100	20 0.67	[8]
MoS_2_ spherical flowers (d ≈ 400 nm) Sn-MoS_2_ spherical flowers (d ≈ 800 nm)	2.4 2.3	53 127	RhB	554	100 100	40 20	[3]

η* is pollutant degradation efficiency after t min of irradiation. d is average size/diameter.

**Table 2:** Representative studies on photodegradation of persistent organic pollutants using MoS_2_-based heterojunction photocatalysts.

Photocatalyst	Heterojun-ction Type	Synthesis Method	Pollutant Conc. (mg/L)	Catalyst Dosage (g/L)	Light Source	η* %	t Min	Ref.
MoS_2_/SnO_2_	II	hydrothermal	MB (8)	2	VIS (150 W Xenon lamp)	99.5	5	[26]
CaO/MoS_2_	Z-scheme	ball milling + calcination (900 °C, 2 h)	MB (57.14)	1	sunlight	70	10	[72]
MoS_2_/ZnO	I	hydrothermal	SMX (20) MX, MB TMP, MG CV	0.8	VIS (1500 W Xenon lamp)	100 100 100 100	30 30 90 120	[24]
MoS_2_/ZnO QDs MoS_2_	I	hydrothermal	TC (20)	0.01	VIS (300 W halogen lamp)	96.5 38.4	120	[23]
MoS_2_/TiO_2_	II	two-step hydrothermal	MB (5)	6.25 cm^2^/ 10 mL MB	VIS (Xenon lamp)	86	180	[1]
MoS_2_/TiO_2_	II	hydrothermal	MB (10)	0.5	VIS (100 W Xe lamp)	74.4	120	[73]
2D MoS_2_/TiO_2_ MoS_2_	I	hydrothermal	RhB (10)	0.3	VIS (125 W Hg lamp)	75 60	25	[22]
MoS_2_/TiO_2_	Z-scheme	hydrothermal	CV (122.4)	0.2	UV (400 W Xe lamp)	94.4	60	[74]
MoS_2_/ZnSe MoS_2_	II	ultrasonication	Levofloxacin (11)	0.3	VIS (500 W Xe lamp)	73.2 29	120	[75]
CuS/MoS_2_	S-scheme p–n	hydrothermal	TC (20)	0.7	VIS, VIS–NIR (LCS-100 solar simulator)	95	30	[13]
CuS/MoS_2_	S-scheme	hydrothermal	HQ (11)	0.5	sunlight	83	240	[76]
CuS/MoS_2_	II	dealloying amorphous Ti-Cu−Mo ribbons in acid solution	MB (10)	0.5	VIS (500 W Xe lamp)	99.9	80	[77]
MoS_2_/CdS	I	solvothermal	NRFX (20)	0.5	VIS (Tungsten halogen lamp)	87.5	25	[30]
MoS_2_/CdS	I	solvothermal	RhB (10)	0.1	VIS (300 W Xe lamp)	83	120	[29]
MoS_2_/CdS	I	solvothermal	RhB (10)	0.5	VIS (300 W Xe lamp)	91.9	60	[78]
MoS_2_/In_2_S_3_	II	hydrothermal	MB (4.8) OTC (0.3)	0.0025	Sunlight (800 W/m^2^)	97.67 76.3	8 40	[32]
MoS_2_/Fe_3_S_4_	-	hydrothermal	TCH (50)	2.5	VIS (300 W Xe lamp)	79.9	60	[79]
MoS_2_/WS_2_ MoS_2_	II	chemical vapor deposition	MB (5)	-	Solar simulator	66.7 43.5	180	[31]
MoS_2_/Zn_0.1_Cd_0.9_S	I	solvothermal	OFX (20)	0.25	VIS (300 W Xe lamp)	90	120	[70]
MoS_2_/ZnCdS	I	photodeposition	TC (30)	0.2	VIS (1 W LED lamp)	75	240	[18]
MoS_2_/ZnFe_2_O_4_	Z-scheme	hydrothermal	MB (10)	0.1	VIS (160 W tungsten-mercury lamp)	92.3	150	[80]
CoNi_2_S_4_/MoS_2_	I	hydrothermal	MB (10)	0.2	VIS (500 W Xe lamp)	100	90	[81]
MoS_2_/FeOCl	II	ultrasonic	TC (50) RhB (10) + 20 μL H_2_O_2_	0.1	VIS (300 W Xe arc lamp)	90 85.4	40 30	[82]
MoS_2_/NiAlFe LTH	S-scheme p–n	hydrothermal	IC (20)	1	VIS (105 W Xe arc lamp)	100	100	[9]
MoS_2_/CaTiO_3_ (CTO)	I	hydrothermal, template-free	RhB (1)	0.033	VIS (15 W LED lamp)	96.88	180	[83]
MoS_2_ /Bi_2_WO_6_	S-scheme p–n	solvothermal	MB (20)	0.2	VIS (1 W LED white light)	97	40	[84]
MoS_2_/Bi_2_WO_6_	S-scheme p–n	solvothermal	TC (10)	0.15	VIS (100 W solar simulator)	96.3	90	[85]
MoS_2_/Bi_4_O_5_Br_2_	S-scheme	In situ mechanical agitation	RhB (10)	0.03	VIS (300 W Xe lamp)	100	24	[57]
MoS_2_/Bi_12_O_17_Cl_2_	S-scheme	ultrasonic assisted	RhB (10)	0.6	VIS (300 W Xe lamp)	92	30	[86]
MoS_2_/g-C_3_N_4_	II	impregnation + calcination	RhB (10)	0.4	VIS (350 W Xe lamp)	99.4	90	[40]
MoS_2_/g-C_3_N_4_	II	hydrothermal	Phenol (10)	0.1	VIS (2.2 kW Xe lamp)	89	20	[39]
MoS_2_/BC	II	hydrothermal	CIP (7)	0.2	VIS (300 W Xe lamp)	92.01	90	[87]
MoS_2_/PPy	II	oxidative polymerization (Ppy) + hydrothermal	MB (5)	0.03	VIS (500 W Xe lamp)	99.3	60	[88]
MoS_2_/SubPc-Br	S-scheme	commercial MoS_2_ calcination with SubPc-Br	CTC (30) CIP (30)	1	VIS (300 WXe lamp)	99.22 98.21	30	[89]
MoS_2_/Cu-MOF	II	hydrothermal	RB (50) CR (50) AZR (50) MO (50) NGB (50)	0.24	sunlight	96.3 80.2 73.7 82.1 63.6	30	[4]
Ag-MoS_2_/COF	Z-scheme	hydrothermal	TC (20)	0.5	VIS (250 W Xe lamp)	90.1	50	[41]
Mn-MoS_2_/rGO	Z-scheme	hydrothermal	RhB (20)	0.25	VIS (60 W compact lamp)	90	240	[90]
MoS_2_/SnO_2_/rGO MoS_2_	II	hydrothermal + ultrasonication	MB	0.2	sunlight	90 51	75	[38]
TiO_2_/RGO/MoS_2_ coatings	-	ultrasonication + dip coating	RhB (4)	1	VIS (LEDs 30,000 lumen)	95	90	[91]
MoS_2_/Fe_2_O_3_/GO MoS_2_	Z-scheme	ball milling + ultrasonication	MB (10)	1	VIS (Xe lamp) sunlight	97.9 88.2	180	[37]
MoS_2_/CdS/CF	II	hydrothermal (MoS_2_) + CBD (CdS)	RHB (10) MB (10) TCH (20)	cloth (4 × 4 cm^2^)	VIS (Xe lamp)	97.3 97.2 55.6	100 70 100	[92]
MoS_2_/ZnO/CNT	Z-scheme	hydrothermal	TC (20)	20	VIS (tungsten light lamp)	95.6	60	[36]
MoS_2_/CuO/gCN	II	hydrothermal+ coprecipitation+ ultrasonication	MO (10) Phenol (10)	0.2	UV (200 W tungsten lamp)	85.14 63.5	35	[93]
AgI/MoS_2_/g-C_3_N_4_	Z-scheme	solvothermal	TCH (10)	0.5	VIS (300 W Xe lamp)	82.8	50	[94]
α-Fe_2_O_3_/MoS_2_/g-C_3_N_4_	Z-scheme	hydrothermal + calcination	RhB (10) MB (10)	0.5	VIS (300 W Xe lamp)	95.5 91.1	80	[95]
CdS/MoS_2_/Mt	I	hydrothermal	TC (20)	40	VIS (LED lamp)	90.03	120	[96]
MoS_2_/Au/CuS	Z-scheme	hydrothermal + in situ chemical reduction	MB (2)	0.25	VIS (PanChum multi-lamp photoreactor)	90.5 day 41 night	60	[69]
MoS_2_/Co_3_O_4_/Cu_2_O	S-scheme	sonication + hydrothermal	MB (30) RhB (30)	1	VIS (500 W halogen lamp)	91 92	100 90	[97]
MoS_2_/TiO_2_/Fe_3_O_4_	-	solvothermal	DCF (5)	0.2	VIS (300 W Xe lamp)	99.6	6	[98]
CT-C-MoS_2_/TiO_2_ textile	I	hydrothermal	RhB (10)	2.5 cm × 5 cm/200 mL RhB	VIS (300 W Xe lamp)	98.8	30	[99]
TiO_2_/Ag/MoS_2_/Ag	-	hydrothermal + Tollen reaction	RhB (20)	0.1	VIS (300 W Xe lamp)	100	60	[100]
ZnS/CdS-Mn/MoS_2_ /TiO_2_	Z-scheme	hydrothermal + successive ionic layer deposition	MO (20) 9-AC (20)	-	VIS (300 W Xe arc lamp)	98 100	100 35	[56]

η* is pollutant degradation efficiency after t min of irradiation.

**Table 3:** Summary comparative table related to the advantages and limitations of MoS_2_-based heterojunction photocatalysts discussed in this review.

Hetero-junction Type	Advantages	Limitations	
		Photocatalytic Mechanism	Photocatalytic System
I	Photogenerated holes are key contributors to the degradation process Photocatalytic performance is supported by the substantial reduction in charge carrier recombination for photocatalysts with high surface areas (porous morphology)	Photogenerated charge carriers are concentrated in only one of the semiconductors, resulting in weak or non-existent improvement in photocatalytic activity	Higher amounts of a photocatalyst could block the effect of other ones in heterojunction (2D MoS_2_/TiO_2_ [22]) Reduced absorption of incident light by photocatalyst due to its lower AQY (MoS_2_/CdS [78]) Pollutant adsorption on the photocatalyst surface or/and the photocatalyst mass reduction after each degradation caused decrease in photocatalyst stability (MoS_2_/CdS [30])
II	Improved charge carrier separation compared with type-1 Enhanced photocatalytic activity for NPs- and QDs-based semiconductors—more active sites on the photocatalyst surface (MoS_2_/ZnO QDs [23]) Good photocatalytic performance (MoS_2_/WS_2_, 66.7% [31]) in direct sunlight irradiation using small amounts of catalyst The design and synthesis of novel high-performance photo-Fenton catalysts	Photogenerated charge carrier transfer and separation are controlled by the narrow band semiconductor (MoS_2_) The active species involved in dye photodegradation are only holes (h^+^) and ∙O_2_^−^ (MoS_2_/g-C_3_N_4_ [40])	The aggregation or collision of photocatalyst particles at higher concentrations reduced photocatalyst active surface area (MoS_2_/Cu-MOF, [4]) Controlling pollutant concentration, higher concentrations could slow degradation rate, blocking the amount of absorbed light (MoS_2_/SnO_2_ [26]) Lower photodegradation efficiencies (39.4–88%) in mixed dye systems under sunlight irradiation (MoS_2_/Cu-MOF, [4]) Synthesis conditions, which influence the structural properties (especially morphology) of the photocatalyst (g-C_3_N_4_/MoS_2_ [40])
Z-scheme	Direct Z-type heterojunction—efficient and versatile pathway to improve photogenerated electron–hole pair separation and direct their transport through composite material (MoS_2_/CaTiO_3_ [83], MoS_2_/ZnO/CNT [36], MoS_2_/Fe_2_O_3_/GO [37]) New insights into the design and synthesis of novel ternary photocatalysts with improved charge separation and stability	Traditional type—redox mediator instability and back reaction, slow charge carrier transfer rate, etc. All-solid-state type—high costs of noble metal mediators	Photocatalyst composition and dosage Dye solution concentration and pH Light source type (UV, VIS, solar simulator, natural sunlight), intensity and distance from the light source (MoS_2_/Fe_2_O_3_/GO [37])
S-scheme	Enhanced photocatalytic performance due to the combination of efficient photogenerated charge carrier transfer and separation across heterojunction interface and high redox capabilities of the individual semiconductors Enhanced photocatalytic activity due to the combination of piezoelectric effect of MoS_2_ nanosheets and photothermal (PT) conversion of both semiconductors in the CuS/MoS_2_ heterojunction photocatalyst (CuS/MoS_2_ [13])	Holes (h^+^) and ∙O_2_^−^ are the active species involved in dye photodegradation (MoS_2_/NiAlFe LTH [9])	Aggregation of MoS_2_ nanosheets and dispersion on the surface of CuS microspheres at higher Mo/Cu mass ratio (100:1) (CuS/MoS_2_ [13]) Experimental conditions effects: pH, light intensity, light source, etc.

## Data Availability

All data are included within the article.

## Abbreviations

The following abbreviations are not included in the manuscript text:

CR	Congo Red	9-AC	9-Anthracene Carboxylic acid
AZR	Alizarin Red	HQ	Hydroquinone
CTC	Chlortetracycline	Mt	Montmorillonite
CIP	Ciprofloxacin	SubPc-Br	Subphthalocyanine bromide
LF	Levofloxacin	PPy	Polypyrrole
DCF	Diclofenac	BC	Biochar
TBC	Thiobencarb		
